# Freedom from infection: enhancing decision-making for malaria elimination

**DOI:** 10.1136/bmjgh-2023-014412

**Published:** 2024-12-07

**Authors:** Luca Nelli, Henry Surendra, Isabel Byrne, Riris Andono Ahmad, Risalia Reni Arisanti, Dyah A S Lesmanawati, Iqbal R F Elyazar, Elin Dumont, Lindsey Wu, Chris Drakeley, Jason Matthiopoulos, Gillian Stresman

**Affiliations:** 1 School of Biodiversity, One Health and Veterinary Medicine, University of Glasgow, Glasgow, UK; 2 Department of Infection Biology, London School of Hygiene and Tropical Medicine, London, UK; 3 Monash University Indonesia, Tangerang Selatan, Indonesia; 4 Oxford University Clinical Research Unit Indonesia, Faculty of Medicine Universitas Indonesia, Jakarta, Indonesia; 5 Centre for Tropical Medicine, Faculty of Medicine, Public Health and Nursing, Universitas Gadjah Mada, Yogyakarta, Indonesia; 6 Department of Biostatistics, Epidemiology and Population Health, Faculty of Medicine, Public Health and Nursing, Universitas Gadjah Mada, Yogyakarta, Indonesia; 7 Department of Epidemiology, College of Public Health, University of South Florida, Tampa, Florida, USA

**Keywords:** Malaria, Health systems evaluation, Mathematical modelling

## Abstract

Assessing elimination of malaria locally requires a surveillance system with high sensitivity and specificity to detect its presence without ambiguity. Currently, the WHO standard criteria of observing the absence of locally acquired cases for 3 consecutive years, combined with a health systems assessment, are used to justify claims of malaria elimination. However, relying on a qualitative framework to support the application of this guideline can lead to early, over-optimistic relaxation of control measures with the potential for resurgence. Overcoming this challenge requires innovative approaches to model the coupled processes of malaria transmission and its clinical observation.

We propose a novel statistical framework based on a state-space model to probabilistically demonstrate the absence of malaria, using routinely collected health system data (which is extensive but inherently imperfect). By simultaneously modelling the expected malaria burden within the population and the probability of detection, we provide a robust estimate of the surveillance system’s sensitivity and the corresponding probability of local elimination (probability of freedom from infection).

Our study reveals a critical limitation of the traditional criterion for declaring malaria elimination, highlighting its inherent bias and potential for misinterpreting ongoing transmission. Such oversight not only misrepresents ongoing transmission but also places communities at risk for larger outbreaks. However, we demonstrate that our integrated approach to data comprehensively addresses this issue, effectively detecting ongoing transmission patterns, even when local reports might suggest otherwise.

Our integrated framework has far-reaching implications for malaria control but also for infectious disease control in general. Our approach addresses the limitations of traditional criteria for declaring freedom from disease and opens the path to true optimisation of the allocation of limited resources. Our findings emphasise the urgent need to reassess existing methods to accurately confirm malaria elimination, and the importance of using comprehensive modelling techniques to continually monitor and maintain the effectiveness of current surveillance systems, enabling decisions grounded in quantitative evidence.

**WHAT IS ALREADY KNOWN ON THIS TOPIC**

**WHAT THIS STUDY ADDS**

- Our study introduces a novel state-space model that concurrently evaluates malaria burden and the likelihood of clinical detection using time series data from multiple health centres.
- This novel framework significantly improves the accuracy of surveillance sensitivity assessments and calculates a reliable probability of local malaria elimination.
- It fills a critical knowledge gap by providing a rigorous, evidence-based tool for decision-making in malaria programmes.

**HOW THIS STUDY MIGHT AFFECT RESEARCH, PRACTICE OR POLICY**

- Our findings indicate that the existing criteria may lead to overly optimistic assessments of malaria elimination, necessitating a comprehensive reassessment of public health strategies.
- The study’s methodologies are readily applicable to other infectious diseases, making it a universal tool for enhancing surveillance effectiveness.
- This will contribute to better informed resource allocation and the development of effective disease control strategies, ultimately shifting current paradigms in public health policy.

## Introduction

 Surveillance, through systematic collection, analysis and interpretation of case data is a core component of any public health programme,^1^ providing evidence to inform the decision-making process and intervention targeting. Data routinely collected at health facilities can quantify the likelihood of a disease circulating in a population. However, although proving the presence of a disease can be unambiguously demonstrated (a single indigenous case would be sufficient), proving the absence of infection with routinely collected health data is challenging because even a single missed infection would lead to incorrect inferences.^2^ Therefore absence can only be probabilistically shown and an approach that can provide a quantitative and unbiased outcome that provides the most accurate answers based on the available data would facilitate decision-making. This problem is routinely faced in veterinary diseases,^3 4^ and pest control,^5^ as well as in human epidemiology,^2^ where passive surveillance contributes the majority of data available for decision-making.

Currently, in the global fight against malaria, surveillance systems are considered as a core intervention.^6^ Many endemic countries have achieved low-transmission or ‘near-elimination’.^7^ However, there are challenges. *Plasmodium* parasites and mosquito vectors are becoming resistant to mitigation measures, the COVID-19 pandemic interrupted both care-seeking and intervention implementation in many regions, and other public health priorities could divert funds away from malaria.^8^,^10^ This makes the need for timely and robust frameworks, with the potential to generate evidence to support evidence-based decision-making for malaria elimination, more pressing.

The WHO defines malaria elimination as the state of having no locally acquired cases for 3 consecutive years and having a surveillance and response system that can adequately prevent the re-establishment of indigenous transmission.^7^ However, unless a population is censused with a perfect diagnostic tool, a time series of reported successive zero cases is not sufficient to ascertain the absence of transmission if it does not account for how effective the surveillance system is at detecting the disease if it exists.^11^

The task of quantifying the likelihood of elimination therefore depends on the simultaneous estimation of (a) the endemic dynamics of the disease (a complex spatiotemporal biological process),^12^ and (b) the sensitivity and specificity of the surveillance system (a complex observation process involving heterogeneous effort and aspects of human behaviour).^13^,^15^

The Surveillance System Sensitivity (SSe), defined as the probability that an infected individual will be detected by the surveillance system,^13^ provides a framework to quantify how effective a surveillance system is. Estimating SSe is typically conducted using scenario tree modelling which relies on the probabilities driving the decision route of an infected person from the development of symptoms to the case being diagnosed.^2 13 16^ The required probabilities at each step can be hard to estimate. Often, data for malaria are either not routinely collected or qualitative (eg, questionnaires completed by healthcare professionals). Thus, parameterising the tree has typically relied on expert opinion, which may be biased and difficult to compare consistently between facilities. In our previous work,^13^ we introduced a data-driven model that estimates these probabilities at each step in the care-seeking cascade. While this approach comprehensively outlines the cascade of possible biases and imprecisions in detecting a case, it does not consider the temporal history of an observation, nor does it factor in spatial associations with supplementary sources of information. The present paper builds on this foundation, further exploring the implications and applications of SSe in the broader context of infectious disease control. By acknowledging the temporal trajectory and spatial associations inherent in disease transmission and detection, we are better positioned to capture the real-world complexities and nuances that affect malaria detection and elimination.

In this paper, we propose a novel formal framework for the context of malaria, based on the original application designed for use in veterinary epidemiology.^2 17 18^ Using routinely collected passive case detection (PCD) data combined with health system interviews, we formulated an analytical approach to reconstruct a state process (ie, the expected degree of malaria transmission in the population) and an observation process (ie, malaria infections being detected by the health system). Ultimately, we provide a time-specific estimate of the probability of freedom from infection (PFree), defined here as the probability that the underlying malaria transmission is below a given elimination threshold.

Our work addresses the challenge of quantifying the latent aspects of both biological and observation processes, which can only be achieved through an integrated analysis of partial and disparate spatial and temporal data. To accomplish this, we developed a statistical inference framework that simultaneously models the following four key components in a computationally efficient manner: spatial patterns, dynamic temporal patterns, an observation process and an epidemiological process. Our framework aims to make the task of decision-making for malaria elimination more manageable and accurate by providing health systems with a tool that better captures the complexities of disease transmission and detection. By doing so, we aim to contribute to more effective resource allocation and ultimately, to the eradication of malaria

## Methods

This study focused on Magelang and Kulon Progo districts in Indonesia, which served as an ideal case study representing a near-elimination setting with varying levels of surveillance, malaria endemicity and transmission connectivity.^13^

**Figure 1:**
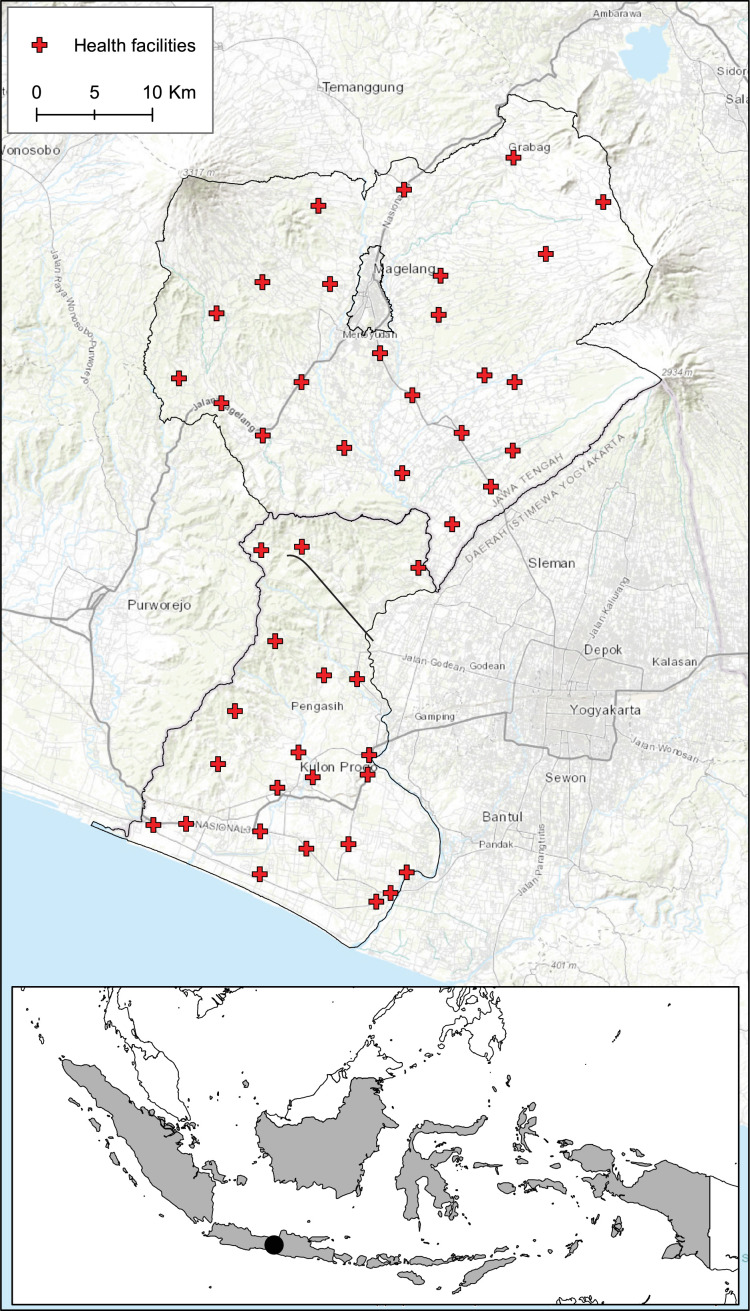
Magelang and Kulon Progo districts in Indonesia showing the locations of health facilities.

Data collection took place between December 2019 and January 2020, using a standardised health systems questionnaire completed via interviews at a subset of 46 health facilities (figure 1). For each health facility, longitudinal data of monthly malaria cases, routinely collected through the passive cases detection system, were collated from January 2017 to December 2019 (36 months). This data included the number of attendees, the estimated catchment population and the number of individuals tested for and diagnosed with malaria.^13^

### Modelling approach

The time series of malaria infections in each catchment area can be described as a combination of a latent state process (true malaria infections arising in a region) and an observation process (malaria cases being detected by the health system). Our model integrates all types of available data to inform difficult-to-estimate parameters and reconstruct partly or wholly latent variables, hence providing a comprehensive analysis of malaria transmission dynamics. These two coupled processes were further connected via spatial (between-catchment) transmission to model all health facility data simultaneously).

#### State process (malaria infections)

We consider the catchment regions of *J*=46 health facilities and a PCD longitudinal series of *I*=36 surveillance months. Irrespective of what is driving the underlying patterns of malaria, we assume that the total number ($M_{i,j}$) of real malaria infections for the *i*th month and the catchment area of the *j*th health centre, is given by the sum of endemic infections (ie, those that originated in the catchment population, $E_{i,j}$) and imported infections (ie, those that originated in any other catchment area within our study site $O_{i,j}$). These components are latent variables, meaning they are not directly observed but are estimated indirectly, through their connections with other, better informed variables in the model:


(1) $$M_{i,j}=E_{i,j}+O_{i,j}$$

We modelled endemic infections as a Poisson process,

,(2) $$E_{i,j}\sim Poisson(\lambda_{E_{i,j}}N_{i,j})$$

where$N_{i,j}$ is the catchment population, and $\lambda_{E_{i,j}}$ is the force of infection related to the proportion of individuals in the population who are expected to have malaria at any given time.

The epidemiological dynamics of endemicity are approximated statistically by a 2nd order autoregressive model, together with an error term ($\varepsilon_{i,j}$) generating extra-Poisson dispersion in the process^19^:


(3) $$log(\lambda_{E_{i,j}})=\alpha_{0\;j}+\alpha_{1}M_{(i-1),j}+\alpha_{2}M_{(i-2),j}+\varepsilon_{i,j},$$

where $exp\;(\alpha_{0})$ (with $\alpha_{0}§amp;lt;0$) determines the expectation of endemic infections at any time when no infections have been observed for two-time lags. The epidemiological parameters $\alpha_{1}$ and $\alpha_{2}$ and the variance of the overdispersion term $\varepsilon_{i,j}$ were the subject of statistical inference during model fitting.

We modelled incoming infections as a Poisson process:

,(4) $$O_{i,j}∼Poisson\left(\lambda_{O_{i,j}}\right)$$

with rate given by

,(5) $$log\left(\lambda_{O_{i,j}}\right)=\;h_{i,j}+\varphi_{i,j}$$

where $\varphi_{i,j}$ is an overdispersion term similar to $\varepsilon_{i,j}$, and $h_{i,j}$ determines the expected incoming infections. We can hypothesise that the incoming malaria infections in each catchment area, are a small proportion of the malaria infections in the nearby areas, weighted by distance. Accordingly, we defined exp⁡(h), which determines the expected rate of incoming case as follows:

,(6) $$exp\left(h_{i,j}\right)=q\sum_{k=1}^{J}M_{i,k}w_{i,k,j}\;/\sum_{k=1}^{J}w_{i,k,j}$$

where

,(7) $$w_{i,k,j}=exp\left(-\gamma D_{k,j}\right)$$

and $D_{k,j}$ is the distance between any pair of health centres, $j\epsilon \left\{1,\ldots ,J\right\}$ and $k\epsilon \left\{1,\ldots ,J\right\}$, γ≥0 is the decay rate determining the connectivity of transmission between j and k, and *q* is the proportion of spillover infections between regions. This parameter regulates the degree of synchrony between catchment regions.

#### Observation process

For the *i*th month and *j*th health centre, we collected data on the number of patients attending the facility ($A_{i,j}$), the number of people reporting fever ($F_{i,j}$), the number of people tested for malaria ($T_{i,j}$) and the number confirmed cases ($C_{i,j}$). These variables are observed directly, with data collected from the health facilities as part of routine surveillance. We structured the observation process as a causal chain of events leading from $M_{i,j}$, the unobserved cases present in each subpopulation to $C_{i,j}$, the malaria cases ultimately being detected by the health system.

### Attending a health facility

We modelled the number of patients attending the health facility as resulting from a Poisson process:

,(8) $$A_{i,j}∼Poisson\left(\lambda_{\alpha_{i,j}}\right)$$

where the rate $\lambda_{\alpha_{i,j}}$ is given by

,(9) $$\lambda_{\alpha_{i,j}}=\left(r_{j}N_{i,j}+P_{CLINICAL}M_{i,j}\right)P_{SEEK_{j}}$$

where$N_{i,j}$ is the catchment population, $r_{j}$ is the background monthly proportion of ill people (ie, people who attend the health facility for all reasons other than malaria) in each catchment population, $P_{CLINICAL}$ is the proportion of symptomatic malaria infections and $P_{{SEEK}_{j}}$ is the probability of care-seeking at each health facility.

Based on our previous finding in the same setting, we assumed the probability of care-seeking, $P_{{SEEK}_{j}}$ in equation (9), varying between health facilities.^13^ In particular, we found that clinics that regularly provide antimalarial drugs are more likely to be attended for care-seeking. In addition, we found a negative effect of antimalarial drug stock-out episodes. We also found a negative effect of the average travel time in the catchment area, indicating that accessibility plays a major role in the probability of care-seeking.^13^ Hence, we modelled ${P_{SEEK}}_{j}$ as


(10) $$logit\;\left(P_{SEEK_{j}}\right)=\;\alpha_{SEEK}+\sum_{k=1}^{S}\beta_{k}X_{j,k},$$

where the linear predictor on the right-hand-side of the expression comprises a set of 𝑆 coefficients 𝛽 and *n* explanatory variables 𝑋 collected at 𝑗th health centre via the survey questionnaires. In particular, based on our previous findings,^13^ we used as explanatory variables whether the health centre provides antimalarial drugs (𝑎𝑛𝑡𝑖𝑚𝑎𝑙_𝑝𝑟𝑜𝑣𝑖𝑑𝑒𝑑), and whether the health centre has experienced a recent stockout of antimalarial drugs (𝑎𝑛𝑡𝑖𝑚𝑎𝑙_𝑠𝑡𝑜𝑐𝑘𝑜𝑢𝑡). These were treated as ‘dummy’ variables and codes as ‘yes’:1 and ‘no’: 0. In addition, we included as covariate the average travel time (𝑡_𝑡𝑖𝑚𝑒) to each health in the catchment area. To calculate this, we followed the approach proposed by Weiss *et al*^20^ and modified by Nelli *et al*^14^ and Ahmad *et al*.^13^

### Showing fever symptoms

We modelled the number of people with fever symptoms as a Poisson process:

,(11) $$F_{i,j}∼Poisson\left(\lambda_{FEV_{i,j}}\right)$$

with the rate $\lambda_{{FEV}_{i,j}}$ given by

,(12) $$\lambda_{FEV_{i,j}}=sr_{j}N_{i,j}+P_{CLINICAL}M_{i,j}$$

where s is the proportion of people with any pyretic disease other than malaria.

### Being tested for malaria

When modelling the number of patients tested for malaria, we assume that this can result from the rate $\lambda_{{FEV}_{i,j}}$, weighted by the probability of being tested $P_{{TEST}_{j}}$, according to:

.(13) $$T_{i,j}∼Poisson\left(P_{TEST_{j}}\lambda_{FEV_{i,j}}\right)$$

### Being diagnosed with malaria

Finally, we modelled the number of patients confirmed with malaria as

,(14) $$C_{i,j}∼Poisson\left(\lambda_{CONF_{i,j}}\right)$$

with the rate $\lambda_{{CONF}_{i,j}}$ defined as

.(15) $$\lambda_{CONF_{i,j}}=P_{CLINICAL}P_{TEST_{j}}M_{i,j}$$

The probability of testing for malaria at health facilities can vary depending on the characteristics of the national malaria monitoring protocols,^21^ and the intrinsic capabilities of the given health facility. Following the approach we have previously proposed,^13^ we modelled ${P_{TEST}}_{j}$ as


(16) $$logit\;\left(P_{TEST_{j}}\right)=\;\alpha_{TEST}+\sum_{k=1}^{V}\beta_{k}X_{j,k}$$

Similar to equation (10), and based again on previous findings,^13^ we used as explanatory variables whether the health centre has functioning microscopy equipment (microscopy_function), whether functioning counting meters were available (microscopy_meters), whether there was a recent stockout of microscopy materials (microscopy_stockout), whether a copy of the national malaria treatment guidelines or standard operating procedures on malaria case management were available for staff in the facility (treatment_sops) and whether the facility received supervisory visits from a district health officer or consultant in the last year (supervision). In addition, to capture the seasonality of malaria cycle, we added the month (1: January to 12: December) with a cubic spline effect. While rapid diagnostic tests (RDTs) are mentioned in the 2013 national guidelines from Indonesia, our previous study found that RDT-based diagnosis was used by only a small proportion of health facilities (0.15), with many facilities experiencing stockouts. Furthermore, variables related to RDT equipment and training did not show a clear effect on the probability of testing in that analysis. As a result, we focused our model on microscopy-based testing, which had a more consistent impact in this context.

Equations (8), (11), (13) and (14) can be generalised to better account for specific data features. For instance, in cases of overdispersion (where the variance exceeds the mean), it may be appropriate to use a negative binomial distribution rather than a Poisson distribution. Additionally, if the number of tested cases ($T_{i,j}$) is known to be a direct proportion of the number of fever cases ($F_{i,j}$), a binomial distribution could be applied, such as $T_{i,j}∼Binomial\left(P_{TEST},F_{i,j}\right)$. However, we chose to use a Poisson distribution instead of a binomial to relax some of the constraints that may be imposed by a cascade of binomial likelihoods, especially given the nature of the data (eg, small errors in medical records can lead to instances where the number of tested cases exceeds the number of reported fever cases).

#### Freedom from infection

We modelled the latent state and observation processes simultaneously in an integrated way. We used the joint posterior distribution of all imputed values of $M_{i,j}$ (across time and regions) to calculate the probability of freedom from infection at a given time point and for a given threshold. We achieved this by calculating what per cent of posterior density at any given time and place falls below the threshold. For example, here we defined PFree as the probability of having achieved elimination at a threshold of less than 1 infection every 10 000 people (ranging between 0 and 1), by measuring how much of the posterior distribution of $M_{i,j}$ is under 1 infection every 10 000.

#### Inference methodology and prior distributions

The analysis was conducted using Bayesian hierarchical model fitting with the program JAGS,^22^ interfaced with the statistical environment R,^23^ via the package *rjags*.^24^ We used three chains and 100 000 iterations to ensure convergence of the MCMC algorithm. To inform the parameters in both the state and the observation processes, we used a mix of expert-based priors and empirical data combined with the semi-qualitative data collected via health centre interviews.^13^ These are described in the following paragraphs and summarised in table 1.

**Table 1:** Description and choice of priors for parameters of the state and the observation process for modelling the probability of freedom from malaria

Process	Parameter	Description	Prior
State	${\alpha_{0}}_{j}$	Average endemic cases	∼Gamma (1,1)
	$\alpha_{1}$	Autoregressive term 1	∼Norm (0,1000)
	$\alpha_{2}$	Autoregressive term 2	∼Norm (0,1000)
	$\varepsilon_{i,j}$	Overdispersion parameter	$∼Norm\;(0,1/\tau_{E})$ $\tau_{E}∼Gamma\;(1,1)$
	$\varphi_{i,j}$	Overdispersion parameter	$∼Norm\;(0,1/\tau_{I})$ $\tau_{I}∼\;Gamma\;(1,1)$
	q	Synchrony parameter	∼Beta (1,10)
	γ	Decay rate	∼Gamma (1,0.1)
Observation	$r_{j}$	Proportion of ill people (non-malaria)	∼Beta (1,10)
	s	Proportion of ill people (non-malaria) with fever	∼Beta (1,10)
	${P_{SEEK}}_{j}$	Probability of care seeking	Modelled as a function of covariates measured at the health facility^13^
	$P_{CLINICAL}$	Probability of symptomatic malaria	∼Beta (50.4,12.6), corresponding to a mean of 0.8 and SD 0.05^25^
	$P_{{TEST}_{i,j}}$	Probability of being tested	Modelled as a function of covariates measured at the health facility^13^

### Parameters and priors of the state process

In equation (3), for the expected endemic cases, we set ${\alpha_{0}}_{j}$ to be negative a priori, so that when the covariates and autocovariates in that expression have small values or effects, the rate of endemic cases should also be close to zero. For $\alpha_{1}$ and $\alpha_{2}$, the terms that regulate the temporal autoregression component of the equation, we chose diffuse, normal priors centred at zero, Norm (0,1000). For the Gaussian term $\varepsilon_{i,j}∼Norm\;(0,1/\tau_{E})$, that controls extra-Poisson dispersion, we used a wide prior for the precision parameter $\tau_{E}∼Gamma\;(1,1)$.

We used naïve priors to model incoming cases’ contributions in equation (5). We hypothesised that the proportion of incoming cases in a catchment area can be only a small proportion of the endemic cases in the surrounding areas, hence we used a Beta (1,10) prior for q in equation (6), corresponding to 0.09±0.08 mean±SD. To model γ, the decay rate in equation (7), we used a Gamma (1,0.1) prior, based on the assumption that the health facilities have a higher chance of being affected by neighbouring facilities at shorter distances (eg, a value of γ=1 would imply that the connectivity drops to 50% of its maximum at 0.7 km distance.

#### Parameters and priors of the observation process

In our model, we allowed for some flexibility in the parameter $r_{j}$, the monthly proportion of ill people (of any non-malaria disease). In particular, we used a Beta (1,10) prior and we indexed it by j, to enable it to vary with the health facility. We based this choice on the assumption that other diseases besides malaria can drive the number of people attending a health facility with different intensities in space.

We assumed $P_{CLINICAL}$, the proportion of symptomatic malaria infections to be fixed in time and space. To model it in equations (12) and (15), we based our prior on previous studies,^25^ and used a Beta (50.4,12.6) prior, corresponding to a mean of 0.8 and SD of 0.05.

For the intercept $\alpha_{SEEK}$ in equation (10), $\alpha_{TEST}$ in equation (16) and all the βs coefficients both equations, we chose diffuse normal priors centred at zero to reflect minimal prior assumptions about the effect of each covariate, allowing the data to primarily inform the estimates.

To model s, the proportion of people ill with any other non-malaria fever-inducing disease in equation (12), we used a Beta (1,10) prior.

## Results

Over the 36 months of observation, 5 96 264 patients showed fever symptoms. Of these, 66 429 were tested for malaria, and 143 malaria cases were detected. Our modelling results show a high degree of heterogeneity of ongoing estimated malaria transmission between the populations in the catchments of the 46 health facilities and system sensitivity.

The online supplemental appendix includes detailed results of the observation and state processes, with the mean of the posterior distributions of all the parameters and their 95% credible intervals (online supplemental table S1). Additionally, we provide a comparison of the prior and posterior distributions (online supplemental figure S2), which show that the data significantly informed the posterior estimates. We also present the full temporal series of all the empirical data (ie, people reporting fever, people tested for malaria, reported malaria cases) of each health facility, the reconstructed underlying malaria transmission and PFree (online supplemental figure S3). Detailed results of the health facility interviews can be found in Ahmad *et al*.^13^

**Figure 2:**
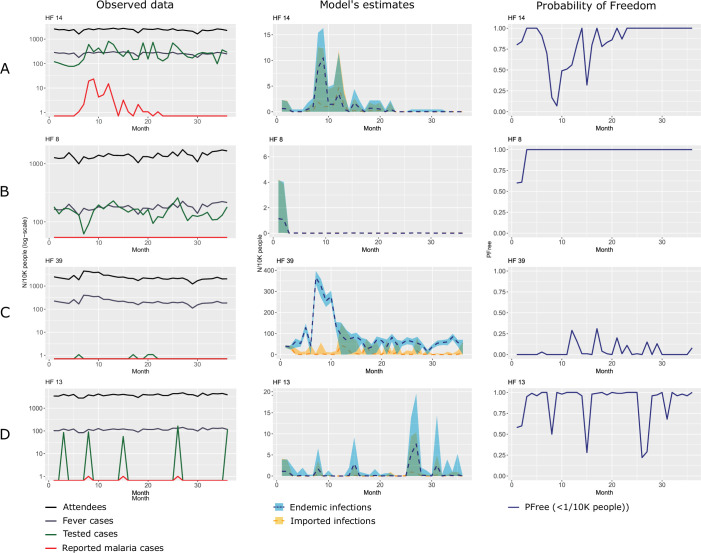
Four examples of possible combinations of malaria transmission and Health System Surveillance in four health facilities in Kulon Progo and Magelang districts (Indonesia): (A) strong surveillance sensitivity and malaria cases reported, (B) strong surveillance sensitivity and zero malaria cases reported, (C) weak surveillance sensitivity and zero malaria cases reported and (D) temporally inconsistent surveillance sensitivity. The first column shows the raw data on a log scale: attendees (black line), reported fever cases (grey line), tested cases (green line) and confirmed malaria cases (red line). The second column shows the reconstruction (blue dashed line) of endemic malaria infections with 95% credible intervals (blue ribbon) and imported infections (orange dashed line and ribbon) according to our model (infections/10K people). The third column shows the probability of freedom from malaria infection (PFree: <1 infection/10,000 people).

Here, we select four examples (figure 2) that illustrate how the method can account for heterogeneities in disease transmission (state process) and diagnostic effort (observation process).

### Example 1: strong surveillance sensitivity and malaria cases reported

In health facilities that exhibit a strong surveillance sensitivity, the actual reported malaria cases should closely mirror the trends in malaria infections reconstructed by the model. Health facility 14 (figure 2A) demonstrates this by showing an outbreak of reported cases around month 9 and then some subsequent outbreaks leading to zero cases being reported towards month 23. In this health facility, the number of tested cases is consistently high and tightly overlaps with the number of fever cases. In examples of this type, the reconstruction of expected malaria infections mirrors this trend. When cases are observed prior to month 23, the estimated PFree, in facility 14, oscillates, and then stabilises at values close to 1 after month 23 when zero cases were reported, and a high SSe was maintained. Here, the zero reported cases observed after month 23 correspond with the expected zero real cases in the population, with a high degree of certainty.

### Example 2: strong surveillance sensitivity and zero malaria cases reported

Other clinics reported zero malaria cases but still exhibited a high SSe. For example, in health facility 8 (figure 2B), zero malaria cases were reported throughout the entire investigation period. However, the number of people tested is again consistently high, therefore, the estimated real cases overlap with the reported ones. In health facilities where a strong SSe is maintained over time and where no cases have been reported for several years, the probability of freedom is achieved more rapidly and is sustained at a high level. In our example, health facility 8 shows this by reaching values of PFree nearing 1 after a few time points and staying high throughout the remainder of the time series.

### Example 3: weak surveillance sensitivity and zero malaria cases reported

In contrast with the previous examples, for some facilities with low SSe that reported no malaria cases, the malaria infections reconstructed by the model was dramatically different. In health facility 39 (figure 2C), for example, we observe an apparent long series of zero reported cases. However, this facility had low testing effort; only one or two people/month were tested for malaria despite having an average of 337 fever cases/month (min=240, max=406). As a result, the estimated number of malaria infections reconstructed by the model oscillated between 0 and 150 infections/10 000 people. In such facilities a high PFree is never achieved, and any increase is not sustained despite no cases being reported.

### Example 4: temporally inconsistent surveillance sensitivity

The case of SSe varying over time was also observed, with fluctuations in testing rates leading to inconsistent surveillance sensitivity throughout the surveillance period. For example, in health facility 13 (figure 2D), the number of individuals tested is very high during certain months—specifically months 4, 9, 15, 26 and 46—while in the remaining months, no tests are conducted. Corresponding to these spikes in testing, a few malaria cases are reported (one case each in months 9, 15 and 26). However, the irregular pattern of testing results in a highly unstable estimate of the underlying malaria transmission. The FFI fluctuates significantly throughout the entire surveillance period. During months of high testing, SSe temporarily improves, but these brief spikes are followed by long periods of no testing, causing uncertainty to rise sharply. This erratic pattern prevents the model from achieving a stable inference on malaria elimination, highlighting the importance of maintaining SSe through time.

#### Spatio-temporal trends

**Figure 3:**
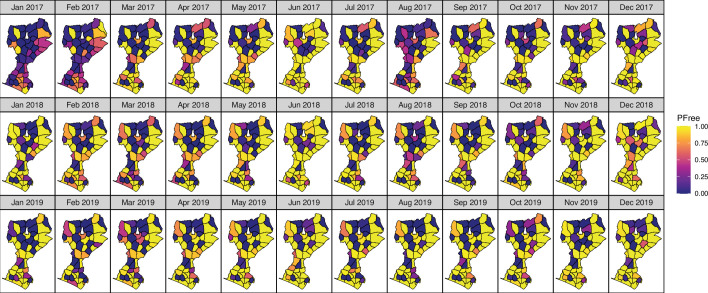
Maps of the estimate of the probability of freedom (PFree) from malaria infection for a given elimination threshold (PFree: <1 infections/10,000 people), in 46 health facilities’ catchment areas in Kulon Progo and Magelang districts (Indonesia) from January 2017 to December 2019.

Spatial-temporal patterns in the estimated PFee per health facility catchment area show a high degree of heterogeneity of PFree under 1 infection/10 000 people (figure 3). Most of the health facilities achieving a consistently high PFree over time are located in Kulon Progo, or the southwestern part of the study area, compared with Magelang. Similarly, most facilities that achieved a high PFree could maintain it throughout the 3-year study duration.

Our results showed that the effect of the connectivity between facilities in the system was minimal, as the parameter q, which quantifies the degrees of synchrony between the clinics, indicated a low influence in the dynamics of the surrounding clinics (mean of posterior q = 0.002; CI 4.19e to 050.009). In addition, the model parameter γ, which quantifies the scale of connectivity, indicated that connectivity in transmission dropped to 50% of its maximum at around 0.21 km distance (mean of posterior of γ =3.27; CI 1.732 to 6.107).

## Discussion

The WHO’s global ambition to eradicate malaria calls for an approach that is both a data-driven and statistically robust. To this end, we present a framework that can generate reliable, evidence-based estimates for the ‘Probability of Freedom’ from malaria infection, or PFree, defined here as the probability of encountering fewer than one malaria infection per 10 000 individuals. Our proposed framework accomplishes this by concurrently modelling both the journey of an infected individual through the healthcare system—from initial infection in the community to detection at a healthcare facility—and the expected number of malaria infections across multiple interconnected health centre catchments, all within an integrated statistical model.

The utility of our framework is twofold. On one hand, it allows for the identification of bottlenecks in the malaria reporting cascade that impact the SSe^13^ and consequently PFree. This kind of nuanced analysis enables the targeted allocation of resources to improve the efficiency of a healthcare system’s surveillance for malaria or other diseases. For example, if a facility is flagged for low SSe and the model identifies low rates of care-seeking or testing as contributing factors, efforts could be channelled into community case management,^26^ or maintaining a consistent stock of testing supplies. On the other hand, the framework serves as a tool for tracking progress toward malaria elimination. It enables the identification of areas where resources should be concentrated, providing internal milestones toward national certification through the option of subnational verification.^7^

While our work is intended to present the base modelling framework and highlight its potential application, it is also designed for adaptability. While we used naïve priors for some parameters in our case study, these can be refined using expert opinion, historical records, or other model outputs. For example, the probability of being tested and the probability of care-seeking were modelled here as simple functions of static covariates at each health centre (eg, the availability of a microscope, trained staff, etc), and basic temporal variables (eg, month) to capture seasonality. However, additional covariates known to influence malaria reporting include socioeconomic status,^27^ ethnicity^28^ or education,^29^ which can be easily added to our base model. Similarly, we used a relatively simple autoregressive model to reconstruct the underlying latent malaria transmission. However, more complex models could be used when modelling the force of infection. For example, we can include some temporal aspects to capture both seasonality, but also some environmental covariates that are known to affect malaria transmission, like temperature,^30^ rainfall^31^ or human activities, if sufficient data is available to inform the models.^32^ This could be further refined by integrating a more intricate mechanistic model. While such a model could potentially offer greater accuracy, it comes at the cost of increased computational complexity. However, an advantage of the current framework is its flexibility in incorporating data from additional surveillance mechanisms. This includes active case detection (ACD) methods such as community cross-sectional surveys, foci investigation or proactive screening.^2 4 33^ If serological data are included in ACD data collection, they can be integrated into our framework, thereby enhancing the precision and accuracy of estimating PFree.^34^

The lack of comprehensive monthly data on different parasite species, like *P. falciparum*, *P. vivax* or others, in this specific case study limited our ability to conduct a more detailed species-specific assessment to confirm elimination and improve surveillance.^35^ Acquiring this level of granular data would enable us to refine our model by incorporating varying probabilities of suspicion for different *Plasmodium* species into our model, considering factors such as geographic location, season and the species-specific sensitivity of RDTs. Additionally, different species have varying risks of recurrence after treatment. For example, *P. vivax*, known for its relapsing nature, presents more complex elimination challenges in comparison to *P. falciparum*. Therefore, the unique biological characteristics of each species need to be carefully considered, especially when multiple species coexist in areas targeted for elimination.

Our model operates on the assumption that a limited percentage of cases recorded in a given health facility are likely to originate from adjacent catchment areas. These are weighted by their relative geographical proximity, thanks to a basic autoregressive framework. However, the model does not distinguish between cases that are locally acquired and those imported from other regions or countries. To enhance the granularity and accuracy of our model, it could be adapted in the future to incorporate importation/relapse status based on travel history or a recent confirmed case, aligning with current WHO recommendations and requirements for malaria-free certification. Future research venues could also fully explore the source-sink dynamics of local parasites, by including molecular data to distinguish between locally acquired and imported cases, and to exclude relapses of imported or introduced *P. vivax* infections. In addition, while our model is currently designed to function at the health facility level, its architecture allows for adaptation to other levels of aggregation, such as district, province, or regional scales, should that prove more useful for the operational needs of specific malaria elimination initiatives.

The case study we examined provided an exemplary setting to validate our model; it typifies a near-elimination scenario characterised by fluctuating levels of surveillance, variability in malaria endemicity, and divergent transmission pathways over time and space. More critically, the study underscores the urgent need for a shift in prevailing approaches to malaria elimination. For instance, Magelang district had been declared malaria-free in 2014, just a few years prior to the period for which our data was collected. Post-certification, there was a noticeable reduction in resources allocated to sustain preventive measures, leading to decreased testing and, consequently, diminished system sensitivity. In a striking development, the district faced a significant malaria outbreak within just a year of its supposed elimination.^36^ Our model retrospectively validates this observation by indicating a reduced PFree across the health facilities in Magelang. Similarly, during the time frame of our study, administrators in the Kulon Progo district invested substantial resources into the healthcare system, with the aim of achieving malaria elimination status from the national programme—a goal that was officially recognised in 2022. Nonetheless, a large outbreak, involving approximately 52 cases, occurred a mere few months post-certification.^37^ Even though Kulon Progo’s health facilities demonstrated generally better sensitivity compared with those in Magelang, our model indicates that malaria elimination had not been consistently and uniformly achieved.

With global funding for malaria control and elimination plateauing since 2015,^8^ the imperative for optimising and maintaining existing surveillance mechanisms has never been greater. In response of this challenge, our model offers a statistically robust and adaptable framework to assess an area’s malaria-free status. While our model is designed to be as adaptable as possible to facilitate programmatic operability, it is clear that future iterations of this work should provide explicit guidelines for practical implementation. Future work should strive to bridge the gap between theoretical robustness and practical applicability, answering the critical questions we have raised. This will ensure that the model will become a functional tool in the fight against malaria and other persistent pathogens.

## Supplementary material

10.1136/bmjgh-2023-014412 online supplemental file 1

## Data Availability

Data are available upon reasonable request.
