# Supplementary material for: Freedom from infection: enhancing decision-making for malaria elimination
Source: BMJ Glob Health. 2024 Dec 7;9(12):e014412. doi: 10.1136/bmjgh-2023-014412 (PMC11628951; doi:10.1136/bmjgh-2023-014412)
Supplement: online supplemental file 1 [file bmjgh-9-12-s001.pdf]

Appendix to:

Nelli et al: Freedom From Infection: Enhancing Decision-Making for Malaria Elimination.

CONTENT

Supplementary Table S1 (posterior distribution of model parameters) ……………………………………….. page 2
Supplementary Figures S2 (Comparison priors vs posteriors)…………………………………………………. page 3
Supplementary Figures S3 (full results for all facilities)……………………………………………………………… page 14

Supplementary Annex 1

Table S1 – Posterior distribution of parameters of the state and the observation process for modelling the probability of freedom from malaria. α_{0_j}: average endemic cases; α_1: autoregressive term 1; α_2: autoregressive term 2; q: synchrony parameter; α_1: autoregressive term 1; γ: decay rate; r_j: proportion of ill people (non-malaria); s: proportion of ill people (non-malaria) with fever; $P_{CLINICAL}$: probability of care seeking; n$_{eff}$: effective sample size.

Parameter	Posterior (mean and 95% credible intervals)	n$_{eff}$	Parameter	Posterior (mean and 95% credible intervals	n$_{eff}$
$\alpha_{0,j}$	-2.65 (-4.362 / -1.051)	1140	r_j	0.26 (0.133 / 0.303)	576
	-18.37 (-23.608 / -13.686)	764		0.25 (0.248 / 0.261)	427
	-2.39 (-2.966 / -1.827)	849		0.04 (0.041 / 0.041)	900
	-2.26 (-3.223 / -1.232)	599		0.17 (0.05 / 0.428)	578
	-1.04 (-1.877 / -0.137)	647		0.1 (0.103 / 0.104)	1100
	-2.89 (-5.128 / -0.539)	1116		0.18 (0.181 / 0.184)	3400
	-16.4 (-21.898 / -11.659)	1055		0.55 (0.534 / 0.568)	835
	-19.28 (-26.73 / -13.906)	1013		0.66 (0.642 / 0.682)	1257
	-4.26 (-6.233 / -2.472)	1183		0.05 (0.051 / 0.051)	2000
	-2.22 (-2.895 / -1.161)	681		0.06 (0.055 / 0.057)	920
	-19.48 (-26.758 / -13.978)	1329		0.37 (0.364 / 0.384)	981
	-16.21 (-21.562 / -12.202)	655		0.05 (0.053 / 0.054)	3400
	-13.11 (-17.78 / -9.308)	563		0.16 (0.158 / 0.171)	1100
	-11 (-14.349 / -7.71)	619		0.52 (0.503 / 0.531)	718
	-14.52 (-19.159 / -10.229)	460		0.66 (0.64 / 0.677)	540
	-1.67 (-2.572 / -0.899)	515		0.12 (0.123 / 0.125)	1114
	-2.54 (-5.698 / -0.068)	893		0.64 (0.191 / 0.868)	577
	-4.54 (-12.805 / -1.15)	668		0.07 (0.046 / 0.123)	1026
	-1.08 (-1.659 / -0.613)	1083		0.1 (0.048 / 0.235)	836
	-1.16 (-1.676 / -0.754)	505		0.08 (0.076 / 0.077)	3400
	-1.7 (-3.201 / -0.137)	1027		0.1 (0.096 / 0.097)	1900
	-1.78 (-2.576 / -1.361)	799		0.03 (0.032 / 0.038)	1400
	-20.22 (-27.587 / -14.769)	1296		0.07 (0.069 / 0.07)	3400
	-17.38 (-22.205 / -13.449)	1016		0.06 (0.06 / 0.06)	3400
	-1.05 (-2.82 / -0.016)	897		0.12 (0.118 / 0.12)	1200
	-1.43 (-2.338 / -0.665)	888		0.13 (0.132 / 0.134)	3000
	-1.41 (-2.659 / -0.021)	996		0.17 (0.167 / 0.169)	1600
	-17.74 (-24.364 / -12.704)	1194		0.27 (0.259 / 0.276)	901
	-16.47 (-20.64 / -13.419)	1123		0.01 (0.008 / 0.008)	2900
	-3.61 (-4.988 / -2.56)	596		0.2 (0.198 / 0.208)	999
	-1.05 (-1.835 / -0.265)	632		0.06 (0.058 / 0.059)	3400
	-1.37 (-2.274 / -0.354)	497		0.06 (0.059 / 0.06)	3400
	-19.16 (-26.317 / -14.127)	1419		0.83 (0.816 / 0.854)	516
	-15.31 (-20.789 / -11.265)	481		0.38 (0.371 / 0.396)	770
	-16.13 (-21.497 / -11.877)	658		0.99 (0.986 / 0.996)	560
	-1.17 (-2.683 / -0.222)	441		0.06 (0.058 / 0.059)	3400
	-1.53 (-2.178 / -1.088)	1113		0.04 (0.038 / 0.038)	1300
	-1.85 (-2.91 / -1.009)	968		0.98 (0.958 / 0.988)	830
	-1.79 (-4.214 / -0.194)	622		0.1 (0.094 / 0.096)	2900
	-1.33 (-1.913 / -0.46)	707		0.05 (0.05 / 0.051)	3400
	-0.65 (-1.819 / -0.008)	449		0.03 (0.032 / 0.032)	1000
	-1.93 (-4.163 / -0.463)	928		0.45 (0.193 / 0.895)	434
	-19.83 (-27.255 / -14.157)	882		0.11 (0.105 / 0.107)	3400
	-2.67 (-3.376 / -1.863)	1072		0.07 (0.067 / 0.068)	520
	-2.81 (-4.483 / -1.263)	561		0.09 (0.094 / 0.095)	1500
	-0.92 (-1.75 / -0.065)	619		0.04 (0.041 / 0.042)	730
	-2.65 (-4.362 / -1.051)	1140		0.26 (0.133 / 0.303)	576
α_1	1.60e-04 (0·878e-05 / 3·143e-04)	1600	s	0·022 (0·021 / 0·023)	514
α_2	-4.23e-04 (-6·278e-04/-2·319e-04)	1800	q	0.002 (4.19e-07 / 0.009)	2600
$P_{CLINICAL}$	0.81 (0.789, 0.847)	3400	γ	3.27 (1.732 / 6.107)	1600

Supplementary Annex 2

Figure S2 – Comparison between Prior and Posterior distribution of parameters of the state and the observation process for modelling the probability of freedom from malaria. α_{0_j}: average endemic cases; α_1: autoregressive term 1; α_2: autoregressive term 2; q: synchrony parameter; α_1: autoregressive term 1; γ: decay rate; r_j: proportion of ill people (non-malaria); s: proportion of ill people (non-malaria) with fever; $P_{CLINICAL}$: probability of care seeking.

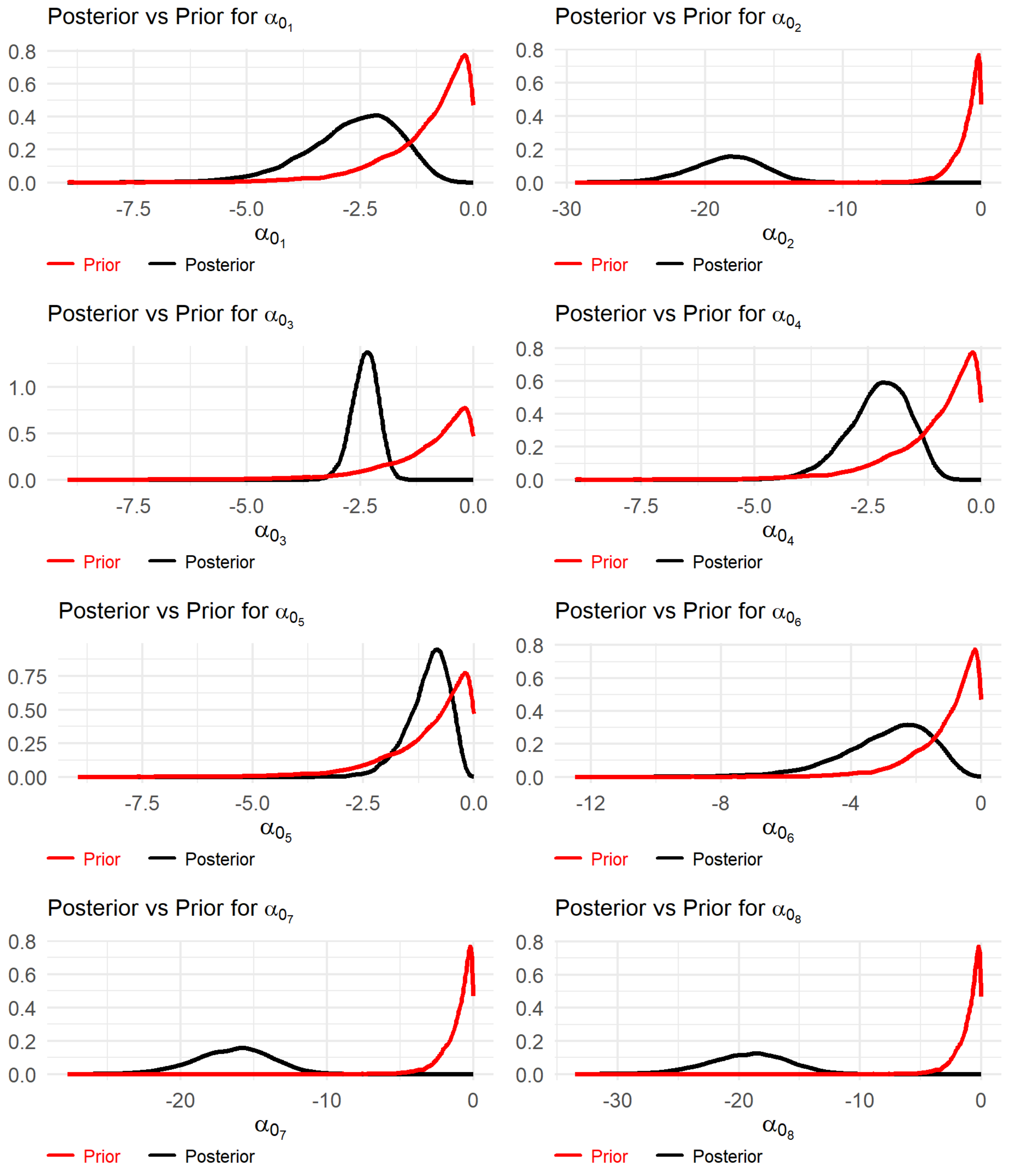

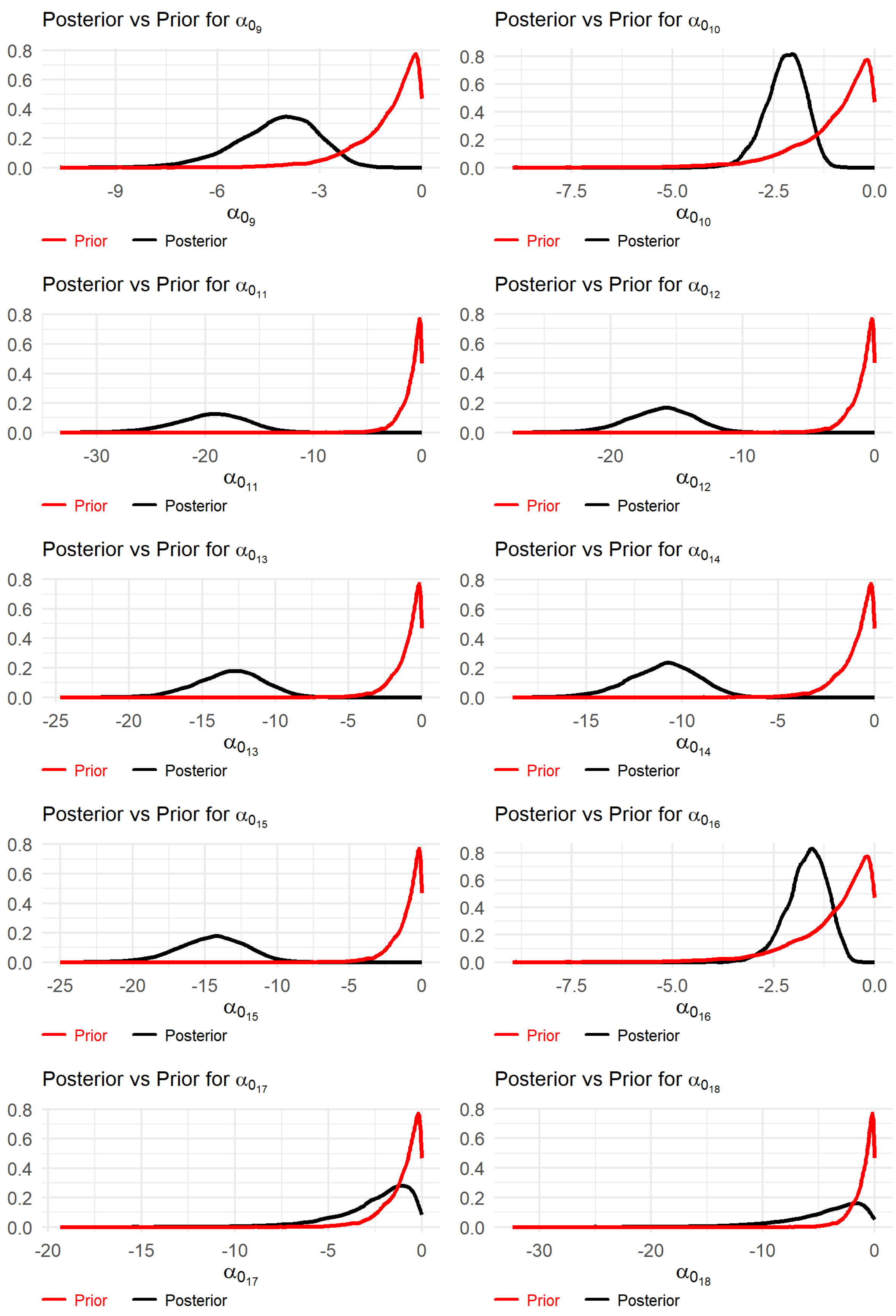
Posterior vs Prior for α_{0_9}
α_{0_9}
Prior
Posterior
Posterior vs Prior for $\alpha_{0_{10}}$
$\alpha_{0_{10}}$
Prior
Posterior
Posterior vs Prior for $\alpha_{0_{11}}$
$\alpha_{0_{11}}$
Prior
Posterior
Posterior vs Prior for $\alpha_{0_{12}}$
$\alpha_{0_{12}}$
Prior
Posterior
Posterior vs Prior for $\alpha_{0_{13}}$
$\alpha_{0_{13}}$
Prior
Posterior
Posterior vs Prior for $\alpha_{0_{14}}$
$\alpha_{0_{14}}$
Prior
Posterior
Posterior vs Prior for $\alpha_{0_{15}}$
$\alpha_{0_{15}}$
Prior
Posterior
Posterior vs Prior for $\alpha_{0_{16}}$
$\alpha_{0_{16}}$
Prior
Posterior
Posterior vs Prior for $\alpha_{0_{17}}$
$\alpha_{0_{17}}$
Prior
Posterior
Posterior vs Prior for $\alpha_{0_{18}}$
$\alpha_{0_{18}}$
Prior
Posterior

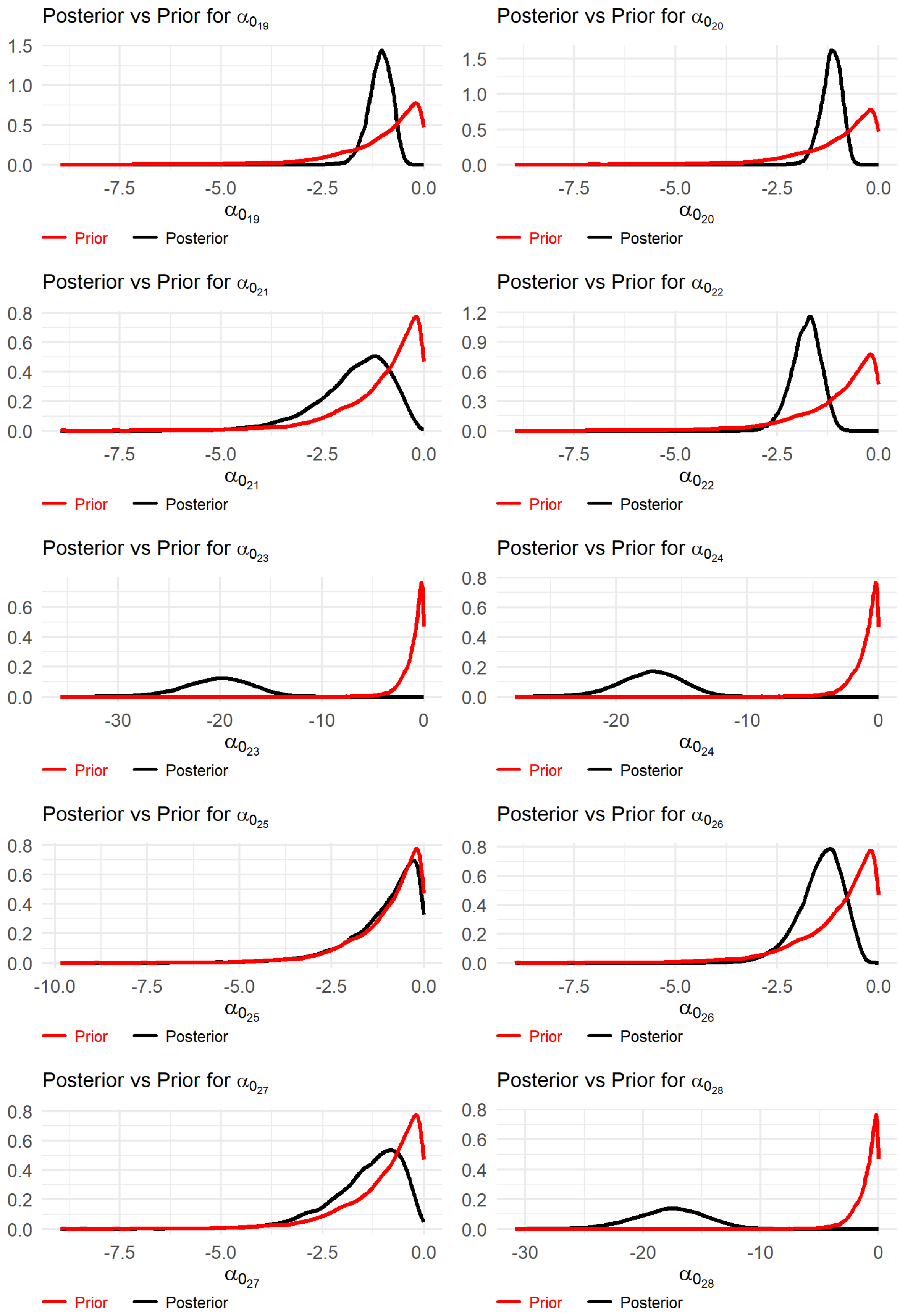
Posterior vs Prior for $\alpha_{0_{19}}$
$\alpha_{0_{19}}$
Prior
Posterior
Posterior vs Prior for $\alpha_{0_{20}}$
$\alpha_{0_{20}}$
Prior
Posterior
Posterior vs Prior for $\alpha_{0_{21}}$
$\alpha_{0_{21}}$
Prior
Posterior
Posterior vs Prior for $\alpha_{0_{22}}$
$\alpha_{0_{22}}$
Prior
Posterior
Posterior vs Prior for $\alpha_{0_{23}}$
$\alpha_{0_{23}}$
Prior
Posterior
Posterior vs Prior for $\alpha_{0_{24}}$
$\alpha_{0_{24}}$
Prior
Posterior
Posterior vs Prior for $\alpha_{0_{25}}$
$\alpha_{0_{25}}$
Prior
Posterior
Posterior vs Prior for $\alpha_{0_{26}}$
$\alpha_{0_{26}}$
Prior
Posterior
Posterior vs Prior for $\alpha_{0_{27}}$
$\alpha_{0_{27}}$
Prior
Posterior
Posterior vs Prior for $\alpha_{0_{28}}$
$\alpha_{0_{28}}$
Prior
Posterior

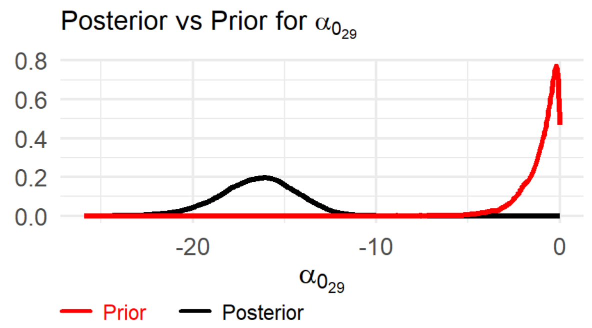
Posterior vs Prior for $\alpha_{0_{29}}$
0.8
0.6
0.4
0.2
0.0
-20
-10
0
$\alpha_{0_{29}}$
Prior
Posterior

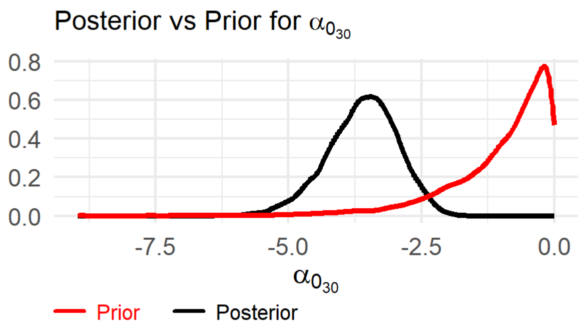
Posterior vs Prior for $\alpha_{0_{30}}$
0.8
0.6
0.4
0.2
0.0
-7.5
-5.0
-2.5
0.0
$\alpha_{0_{30}}$
Prior
Posterior

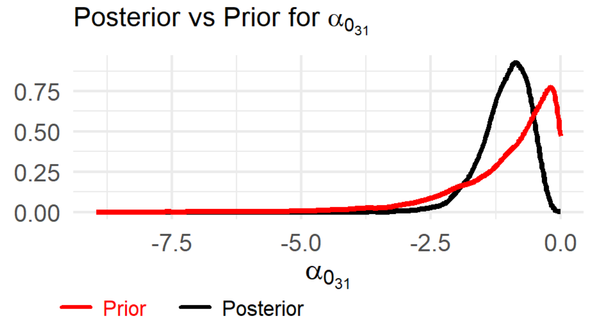
Posterior vs Prior for $\alpha_{0_{31}}$
0.75
0.50
0.25
0.00
-7.5
-5.0
-2.5
0.0
$\alpha_{0_{31}}$
Prior
Posterior

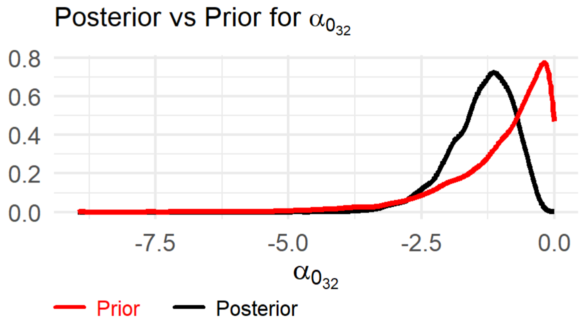
Posterior vs Prior for $\alpha_{0_{32}}$
0.8
0.6
0.4
0.2
0.0
-7.5
-5.0
-2.5
0.0
$\alpha_{0_{32}}$
Prior
Posterior

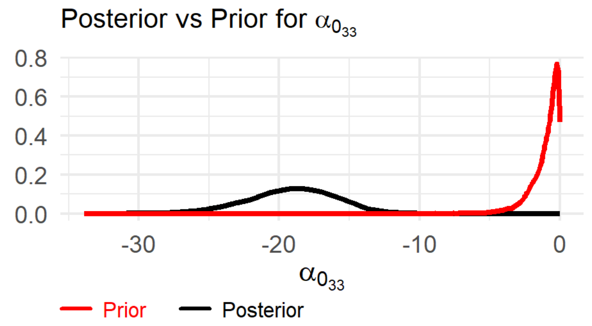
Posterior vs Prior for $\alpha_{0_{33}}$
0.8
0.6
0.4
0.2
0.0
-30
-20
-10
0
$\alpha_{0_{33}}$
Prior
Posterior

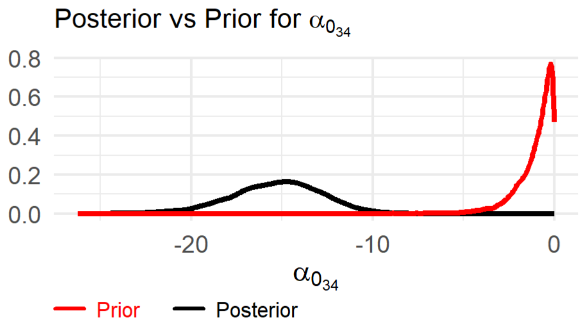
Posterior vs Prior for $\alpha_{0_{34}}$
0.8
0.6
0.4
0.2
0.0
-20
-10
0
$\alpha_{0_{34}}$
Prior
Posterior

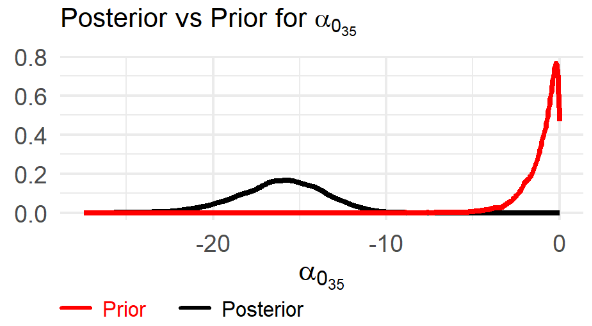
Posterior vs Prior for $\alpha_{0_{35}}$
0.8
0.6
0.4
0.2
0.0
-20
-10
0
$\alpha_{0_{35}}$
Prior
Posterior

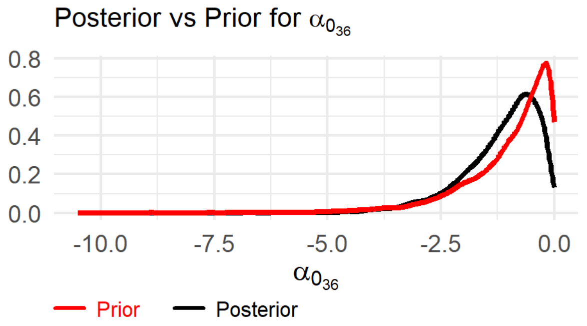
Posterior vs Prior for $\alpha_{0_{36}}$
0.8
0.6
0.4
0.2
0.0
-10.0
-7.5
-5.0
-2.5
0.0
$\alpha_{0_{36}}$
Prior
Posterior

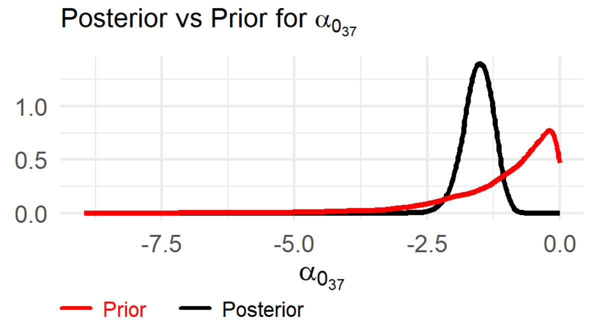
Posterior vs Prior for $\alpha_{0_{37}}$
1.0
0.5
0.0
-7.5
-5.0
-2.5
0.0
$\alpha_{0_{37}}$
Prior
Posterior

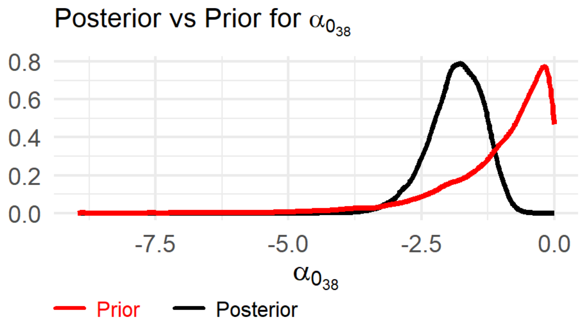
Posterior vs Prior for $\alpha_{0_{38}}$
0.8
0.6
0.4
0.2
0.0
-7.5
-5.0
-2.5
0.0
$\alpha_{0_{38}}$
Prior
Posterior

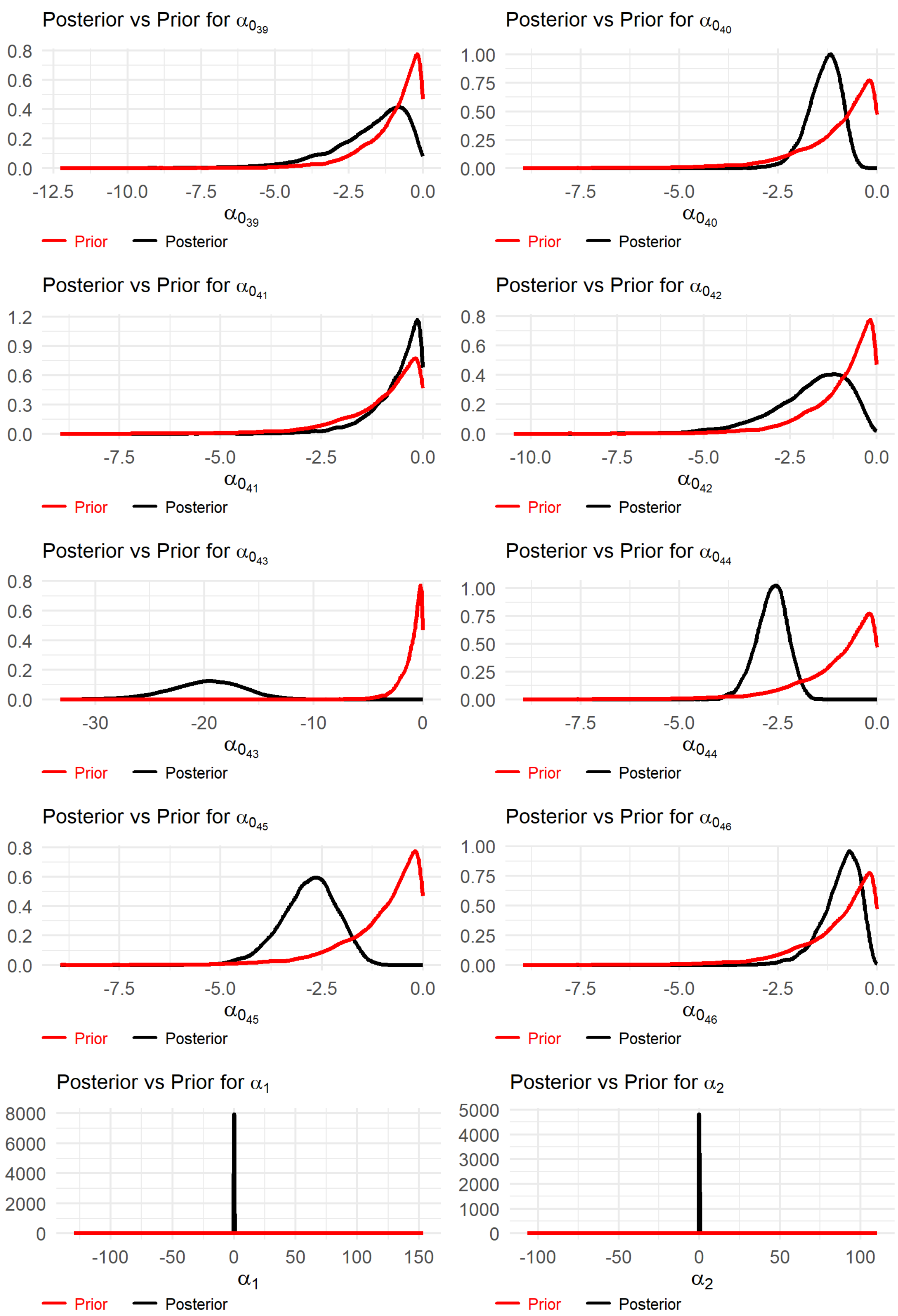
Posterior vs Prior for $\alpha_{0_{39}}$
0.8
0.6
0.4
0.2
0.0
-12.5
-10.0
-7.5
-5.0
-2.5
0.0
$\alpha_{0_{39}}$
Prior
Posterior
Posterior vs Prior for $\alpha_{0_{40}}$
1.00
0.75
0.50
0.25
0.00
-7.5
-5.0
-2.5
0.0
$\alpha_{0_{40}}$
Prior
Posterior
Posterior vs Prior for $\alpha_{0_{41}}$
1.2
0.9
0.6
0.3
0.0
-7.5
-5.0
-2.5
0.0
$\alpha_{0_{41}}$
Prior
Posterior
Posterior vs Prior for $\alpha_{0_{42}}$
0.8
0.6
0.4
0.2
0.0
-10.0
-7.5
-5.0
-2.5
0.0
$\alpha_{0_{42}}$
Prior
Posterior
Posterior vs Prior for $\alpha_{0_{43}}$
0.8
0.6
0.4
0.2
0.0
-30
-20
-10
0
$\alpha_{0_{43}}$
Prior
Posterior
Posterior vs Prior for $\alpha_{0_{44}}$
1.00
0.75
0.50
0.25
0.00
-7.5
-5.0
-2.5
0.0
$\alpha_{0_{44}}$
Prior
Posterior
Posterior vs Prior for $\alpha_{0_{45}}$
0.8
0.6
0.4
0.2
0.0
-7.5
-5.0
-2.5
0.0
$\alpha_{0_{45}}$
Prior
Posterior
Posterior vs Prior for $\alpha_{0_{46}}$
1.00
0.75
0.50
0.25
0.00
-7.5
-5.0
-2.5
0.0
$\alpha_{0_{46}}$
Prior
Posterior
Posterior vs Prior for α_1
8000
6000
4000
2000
0
-100
-50
0
50
100
150
α_1
Prior
Posterior
Posterior vs Prior for α_2
5000
4000
3000
2000
1000
0
-100
-50
0
50
100
α_2
Prior
Posterior

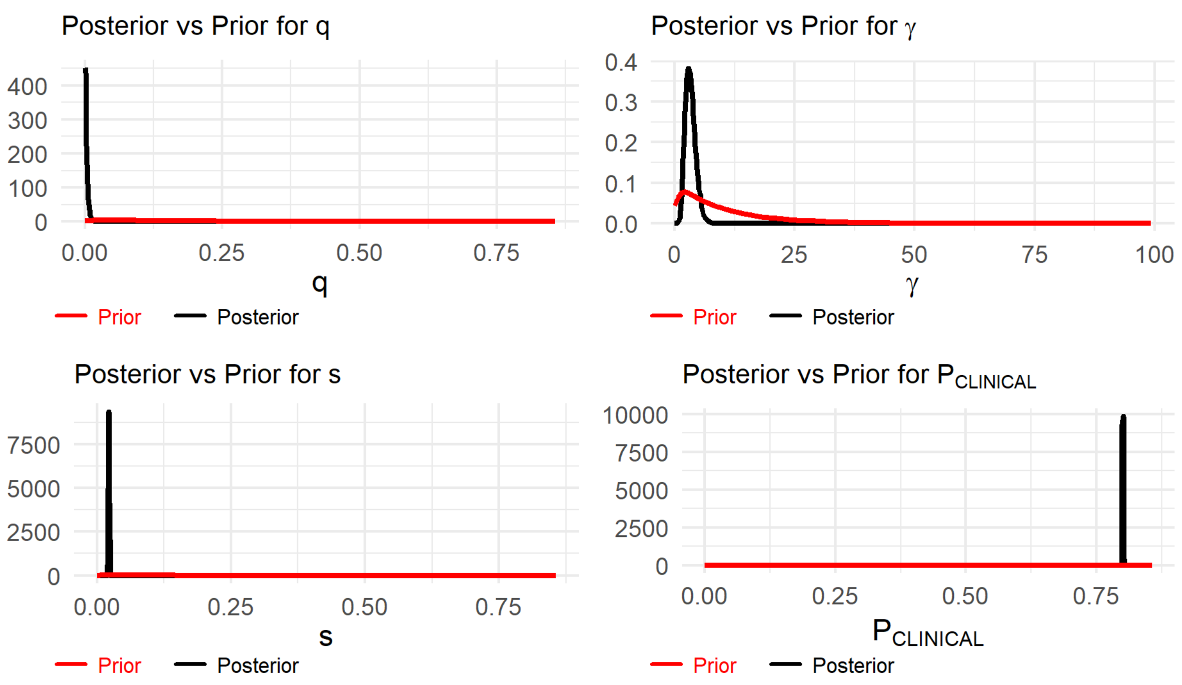
Posterior vs Prior for q
400
300
200
100
0
0.00
0.25
0.50
0.75
q
Prior
Posterior
Posterior vs Prior for γ
0.4
0.3
0.2
0.1
0.0
0
25
50
75
100
γ
Prior
Posterior
Posterior vs Prior for s
7500
5000
2500
0
0.00
0.25
0.50
0.75
s
Prior
Posterior
Posterior vs Prior for P CLINICAL
10000
7500
5000
2500
0
0.00
0.25
0.50
0.75
P CLINICAL
Prior
Posterior

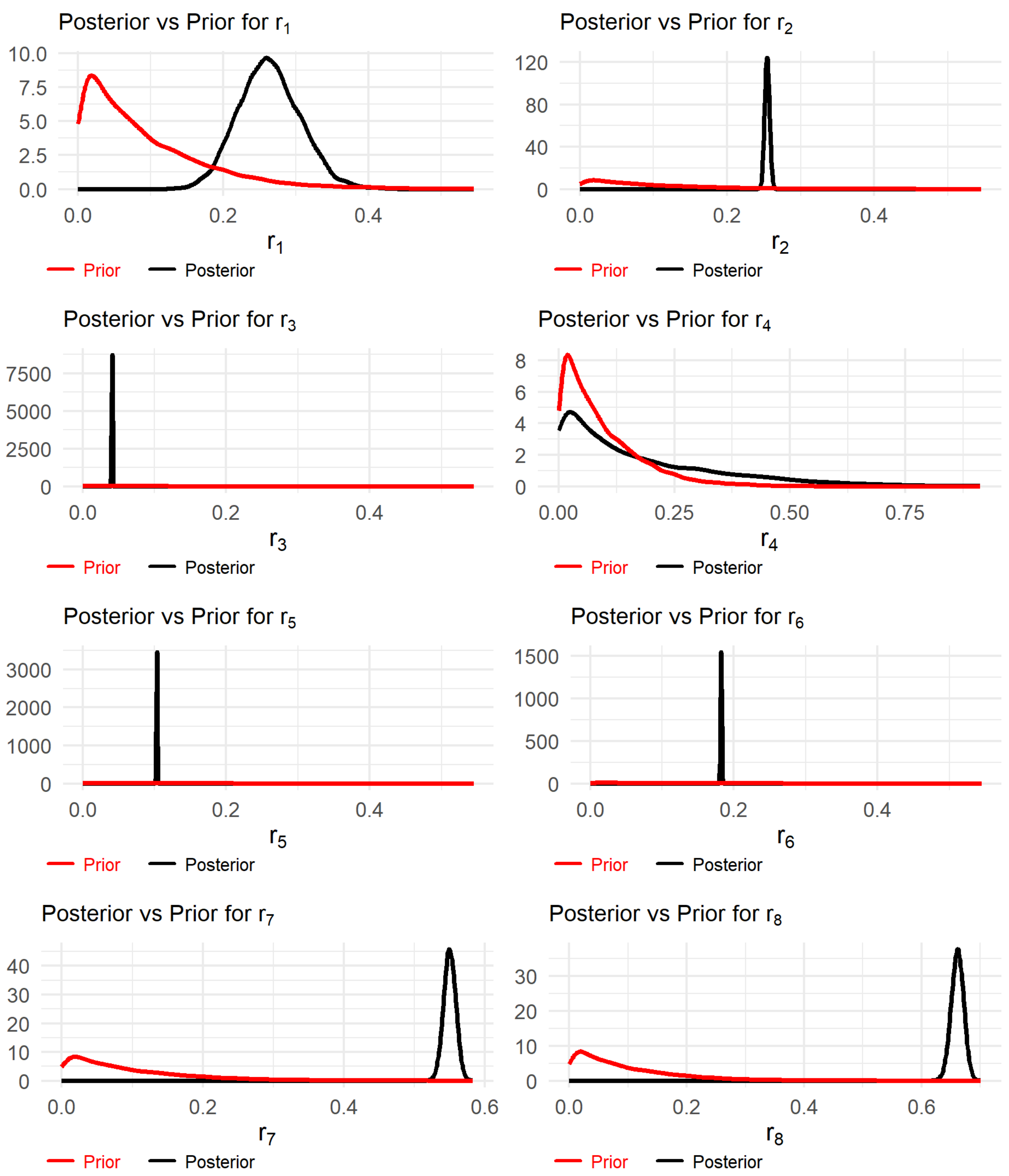
Posterior vs Prior for r_1
10.0
7.5
5.0
2.5
0.0
0.0
0.2
0.4
r_1
Prior
Posterior
Posterior vs Prior for r_2
120
80
40
0
0.0
0.2
0.4
r_2
Prior
Posterior
Posterior vs Prior for r_3
7500
5000
2500
0
0.0
0.2
0.4
r_3
Prior
Posterior
Posterior vs Prior for r_4
8
6
4
2
0
0.00
0.25
0.50
0.75
r_4
Prior
Posterior
Posterior vs Prior for r_5
3000
2000
1000
0
0.0
0.2
0.4
r_5
Prior
Posterior
Posterior vs Prior for r_6
1500
1000
500
0
0.0
0.2
0.4
r_6
Prior
Posterior
Posterior vs Prior for r_7
40
30
20
10
0
0.0
0.2
0.4
0.6
r_7
Prior
Posterior
Posterior vs Prior for r_8
30
20
10
0
0.0
0.2
0.4
0.6
r_8
Prior
Posterior

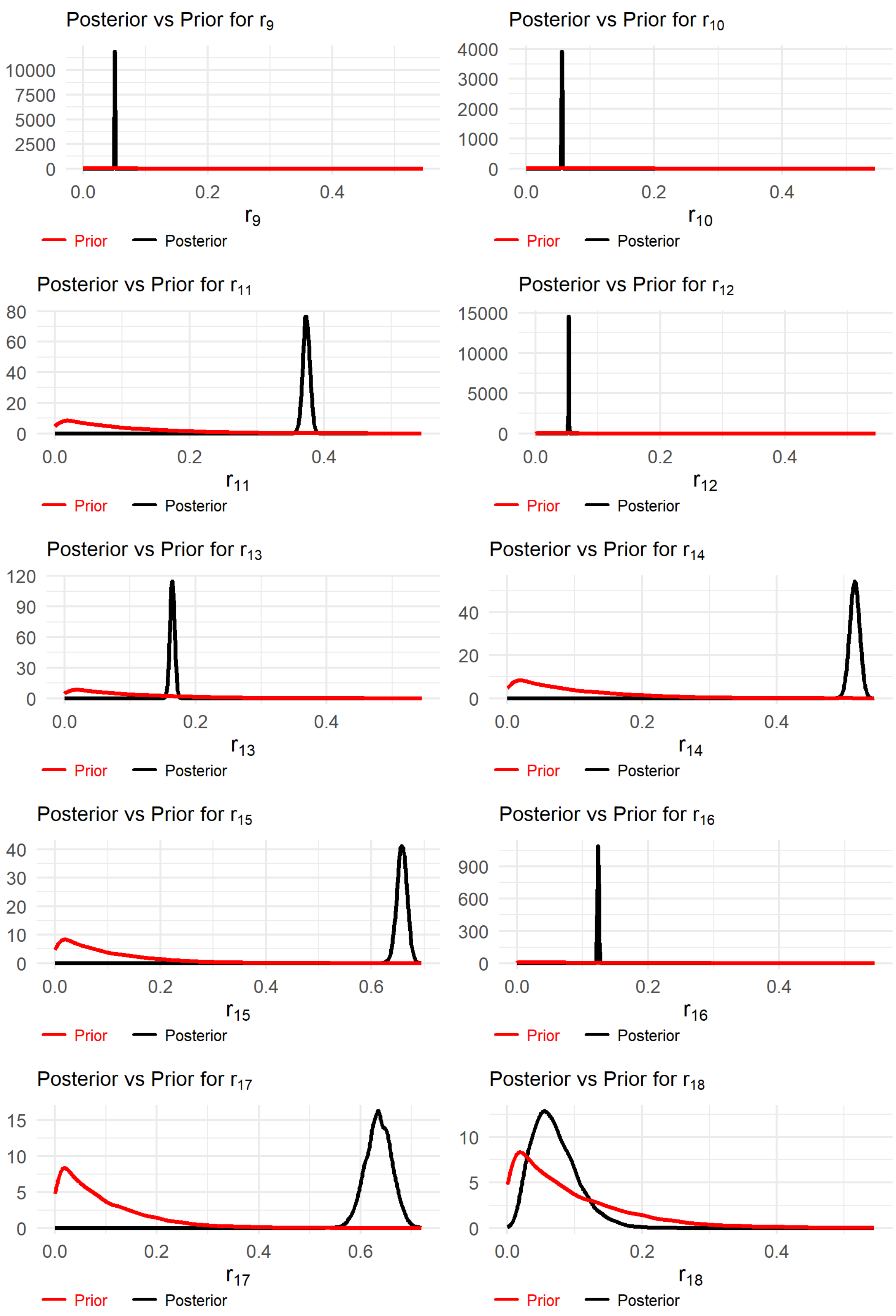
Posterior vs Prior for r9
10000
7500
5000
2500
0
0.0
0.2
0.4
r9
Prior
Posterior
Posterior vs Prior for r10
4000
3000
2000
1000
0
0.0
0.2
0.4
r10
Prior
Posterior
Posterior vs Prior for r11
80
60
40
20
0
0.0
0.2
0.4
r11
Prior
Posterior
Posterior vs Prior for r12
15000
10000
5000
0
0.0
0.2
0.4
r12
Prior
Posterior
Posterior vs Prior for r13
120
90
60
30
0
0.0
0.2
0.4
r13
Prior
Posterior
Posterior vs Prior for r14
40
20
0
0.0
0.2
0.4
r14
Prior
Posterior
Posterior vs Prior for r15
40
30
20
10
0
0.0
0.2
0.4
0.6
r15
Prior
Posterior
Posterior vs Prior for r16
900
600
300
0
0.0
0.2
0.4
r16
Prior
Posterior
Posterior vs Prior for r17
15
10
5
0
0.0
0.2
0.4
0.6
r17
Prior
Posterior
Posterior vs Prior for r18
10
5
0
0.0
0.2
0.4
r18
Prior
Posterior

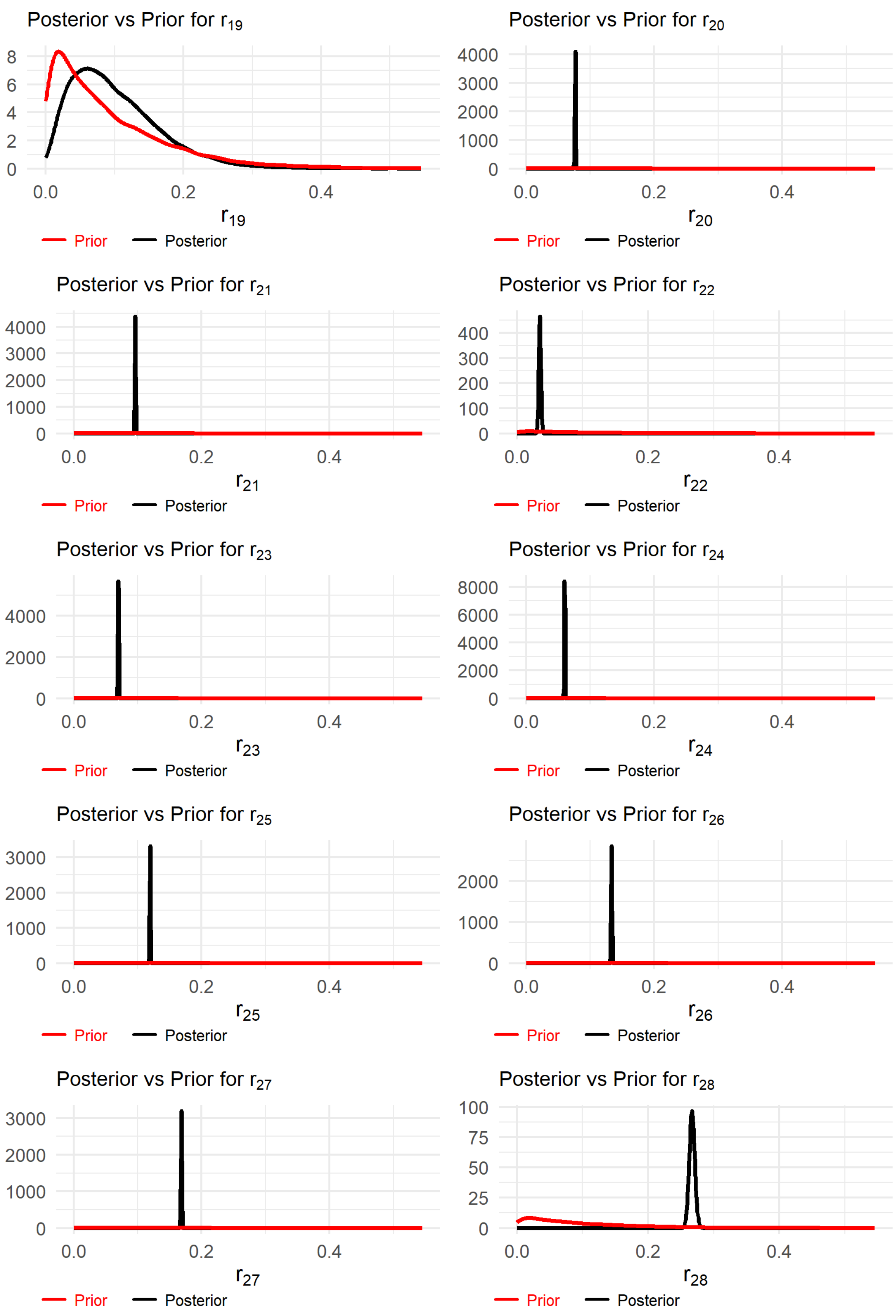

Posterior vs Prior for r_{19}
r_{19}
Prior
Posterior
Posterior vs Prior for r_{20}
r_{20}
Prior
Posterior
Posterior vs Prior for r_{21}
r_{21}
Prior
Posterior
Posterior vs Prior for r_{22}
r_{22}
Prior
Posterior
Posterior vs Prior for r_{23}
r_{23}
Prior
Posterior
Posterior vs Prior for r_{24}
r_{24}
Prior
Posterior
Posterior vs Prior for r_{25}
r_{25}
Prior
Posterior
Posterior vs Prior for r_{26}
r_{26}
Prior
Posterior
Posterior vs Prior for r_{27}
r_{27}
Prior
Posterior
Posterior vs Prior for r_{28}
r_{28}
Prior
Posterior

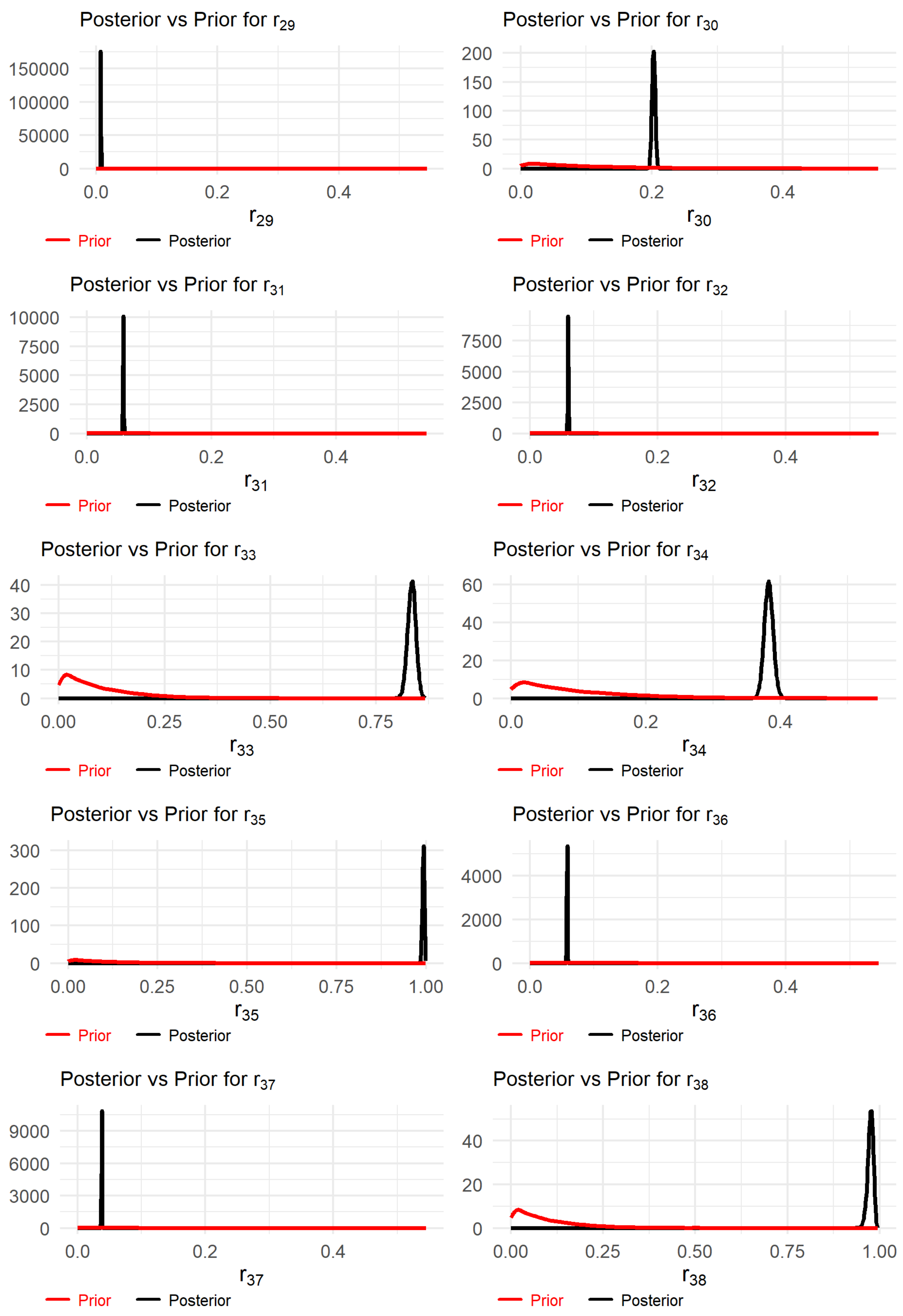

Posterior vs Prior for r_{29}
150000
100000
50000
0
0.0
0.2
0.4
r_{29}
Prior
Posterior
Posterior vs Prior for r_{30}
200
150
100
50
0
0.0
0.2
0.4
r_{30}
Prior
Posterior
Posterior vs Prior for r_{31}
10000
7500
5000
2500
0
0.0
0.2
0.4
r_{31}
Prior
Posterior
Posterior vs Prior for r_{32}
7500
5000
2500
0
0.0
0.2
0.4
r_{32}
Prior
Posterior
Posterior vs Prior for r_{33}
40
30
20
10
0
0.00
0.25
0.50
0.75
r_{33}
Prior
Posterior
Posterior vs Prior for r_{34}
60
40
20
0
0.0
0.2
0.4
r_{34}
Prior
Posterior
Posterior vs Prior for r_{35}
300
200
100
0
0.00
0.25
0.50
0.75
1.00
r_{35}
Prior
Posterior
Posterior vs Prior for r_{36}
4000
2000
0
0.0
0.2
0.4
r_{36}
Prior
Posterior
Posterior vs Prior for r_{37}
9000
6000
3000
0
0.0
0.2
0.4
r_{37}
Prior
Posterior
Posterior vs Prior for r_{38}
40
20
0
0.00
0.25
0.50
0.75
1.00
r_{38}
Prior
Posterior

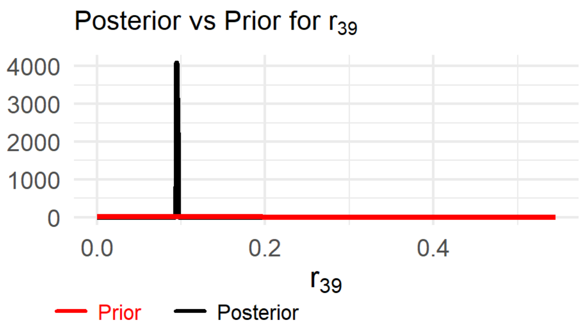
Posterior vs Prior for r39
4000
3000
2000
1000
0
0.0
0.2
0.4
r39
Prior
Posterior

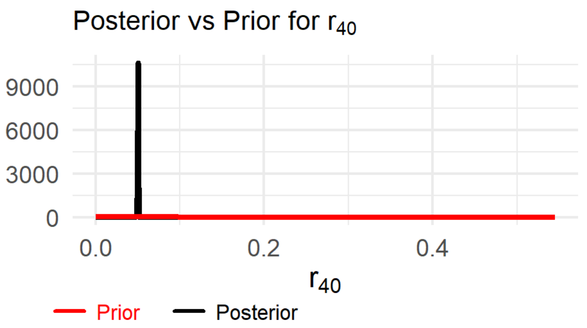
Posterior vs Prior for r40
9000
6000
3000
0
0.0
0.2
0.4
r40
Prior
Posterior

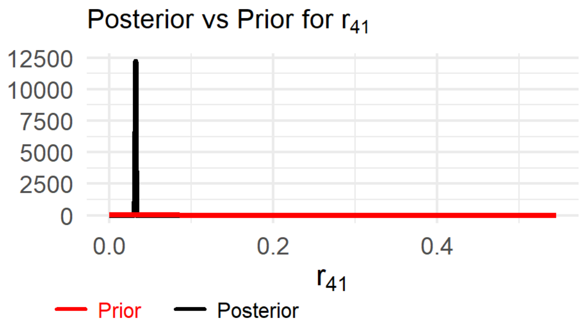
Posterior vs Prior for r41
12500
10000
7500
5000
2500
0
0.0
0.2
0.4
r41
Prior
Posterior

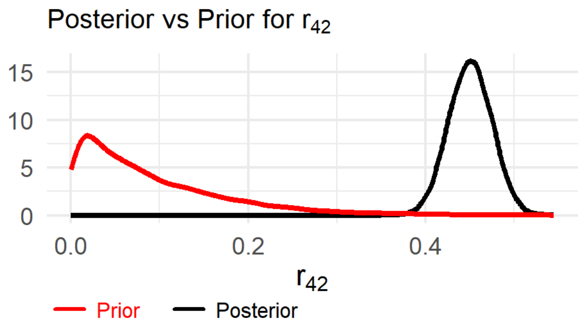
Posterior vs Prior for r42
15
10
5
0
0.0
0.2
0.4
r42
Prior
Posterior

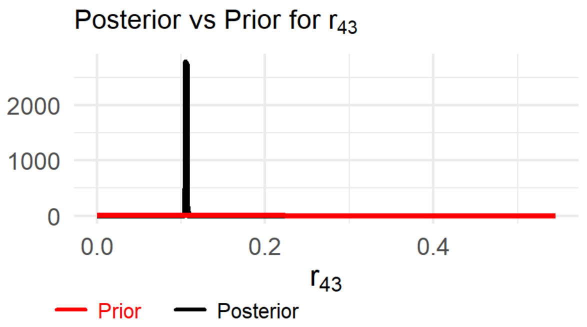
Posterior vs Prior for r43
2000
1000
0
0.0
0.2
0.4
r43
Prior
Posterior

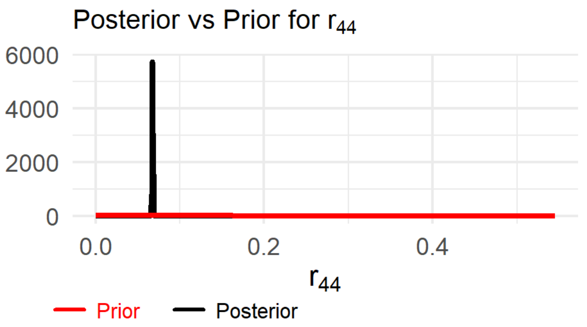
Posterior vs Prior for r44
6000
4000
2000
0
0.0
0.2
0.4
r44
Prior
Posterior

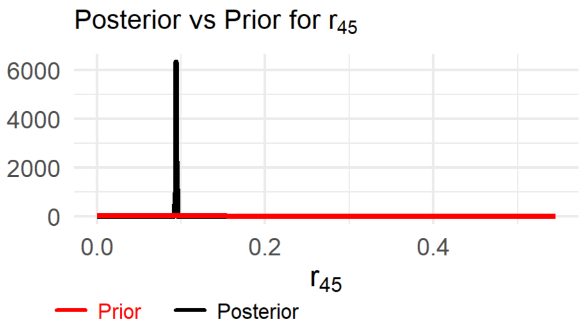
Posterior vs Prior for r45
6000
4000
2000
0
0.0
0.2
0.4
r45
Prior
Posterior

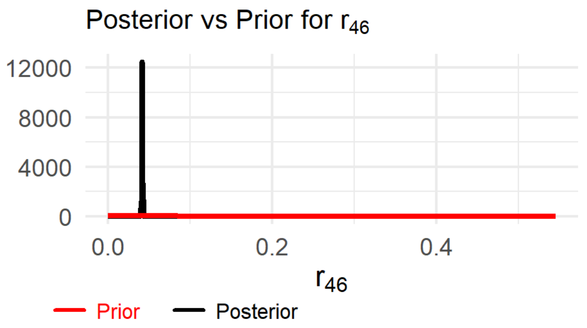
Posterior vs Prior for r46
12000
8000
4000
0
0.0
0.2
0.4
r46
Prior
Posterior

Supplementary Annex 3

Figure S3 – Raw data and model's estimates of malaria transmission and Health System Surveillance in 46 health facilities in Kulon Progo and Magelang districts. The first column shows the raw data on a log scale: attendees (black line), reported fever cases (grey line), tested cases (green line) and confirmed malaria cases (red line). The second column shows the reconstruction (blue dashed line) of endemic malaria infections with 95% credible intervals (blue ribbon) and imported infections (orange dashed line and ribbon) according to our model (cases/10K people). The third column shows the probability of freedom from malaria infection (PFree: <1 infection/10,000 people).

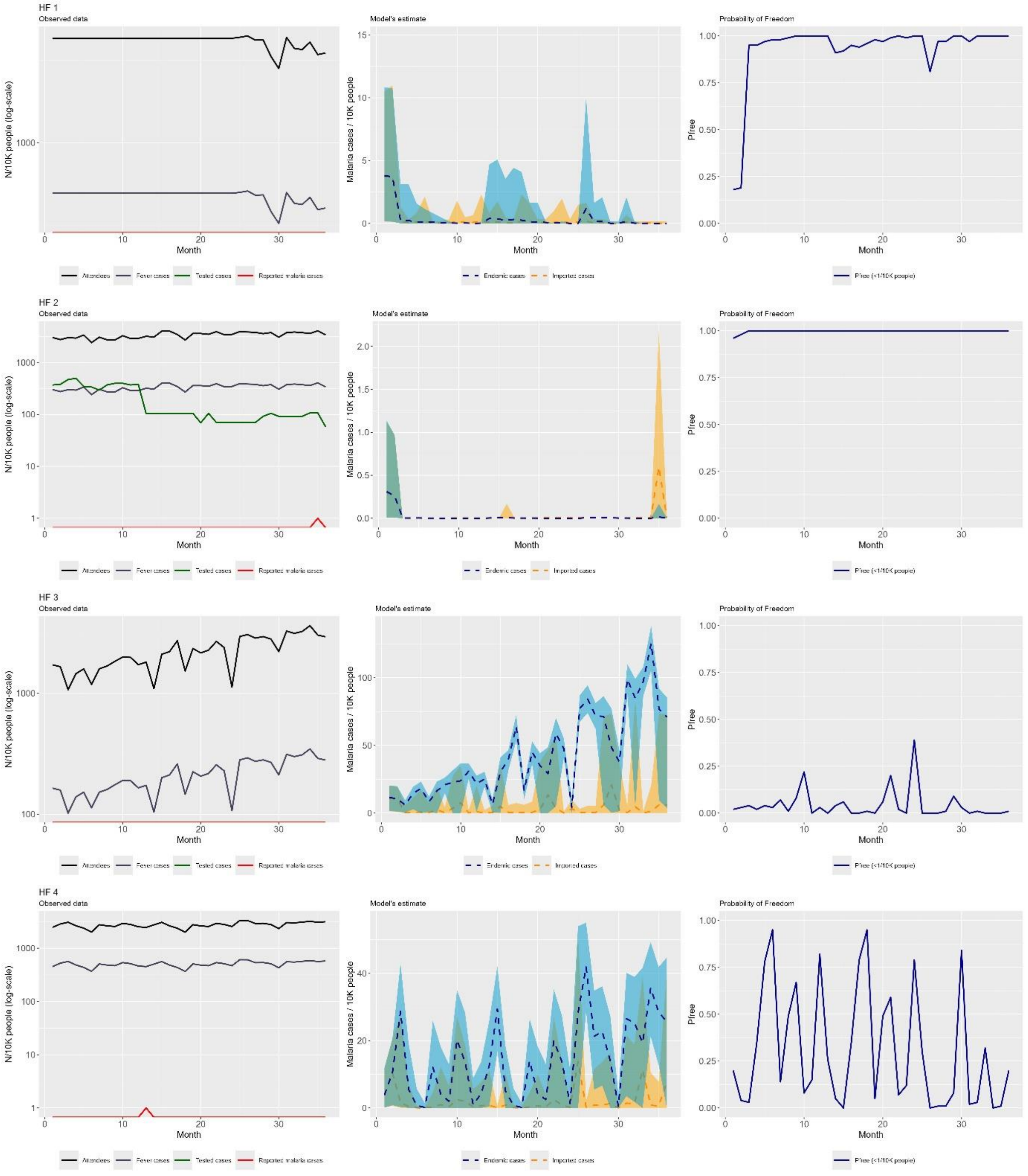

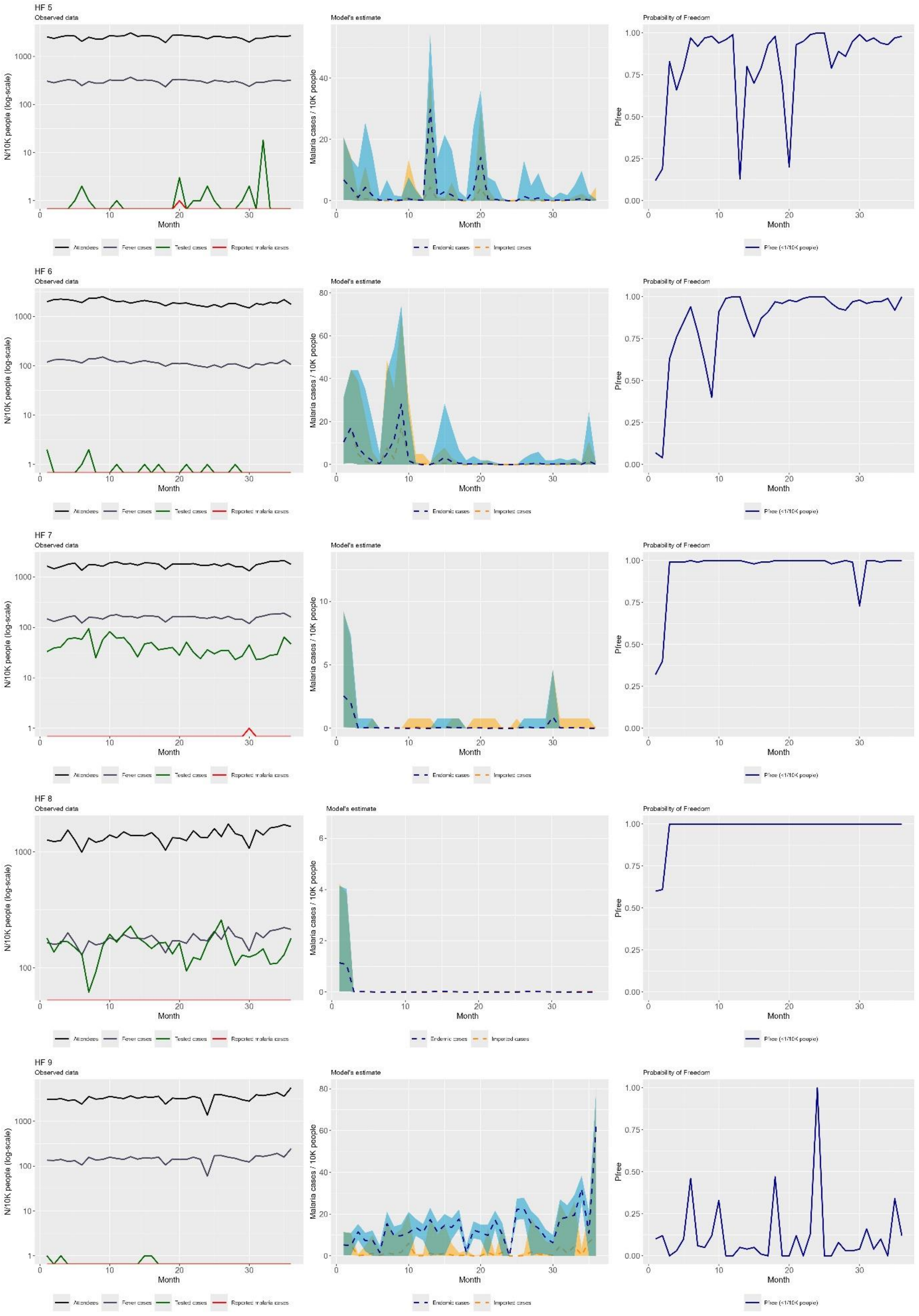
HF 5
Observed data
Model's estimate
Probability of Freedom
HF 6
Observed data
Model's estimate
Probability of Freedom
HF 7
Observed data
Model's estimate
Probability of Freedom
HF 8
Observed data
Model's estimate
Probability of Freedom
HF 9
Observed data
Model's estimate
Probability of Freedom
N/10K people (log-scale)
Malaria cases / 10K people
Pfree
Month
Attendees
Fever cases
Tested cases
Reported malaria cases
Endemic cases
Imported cases
Pfree (<1/10K people)

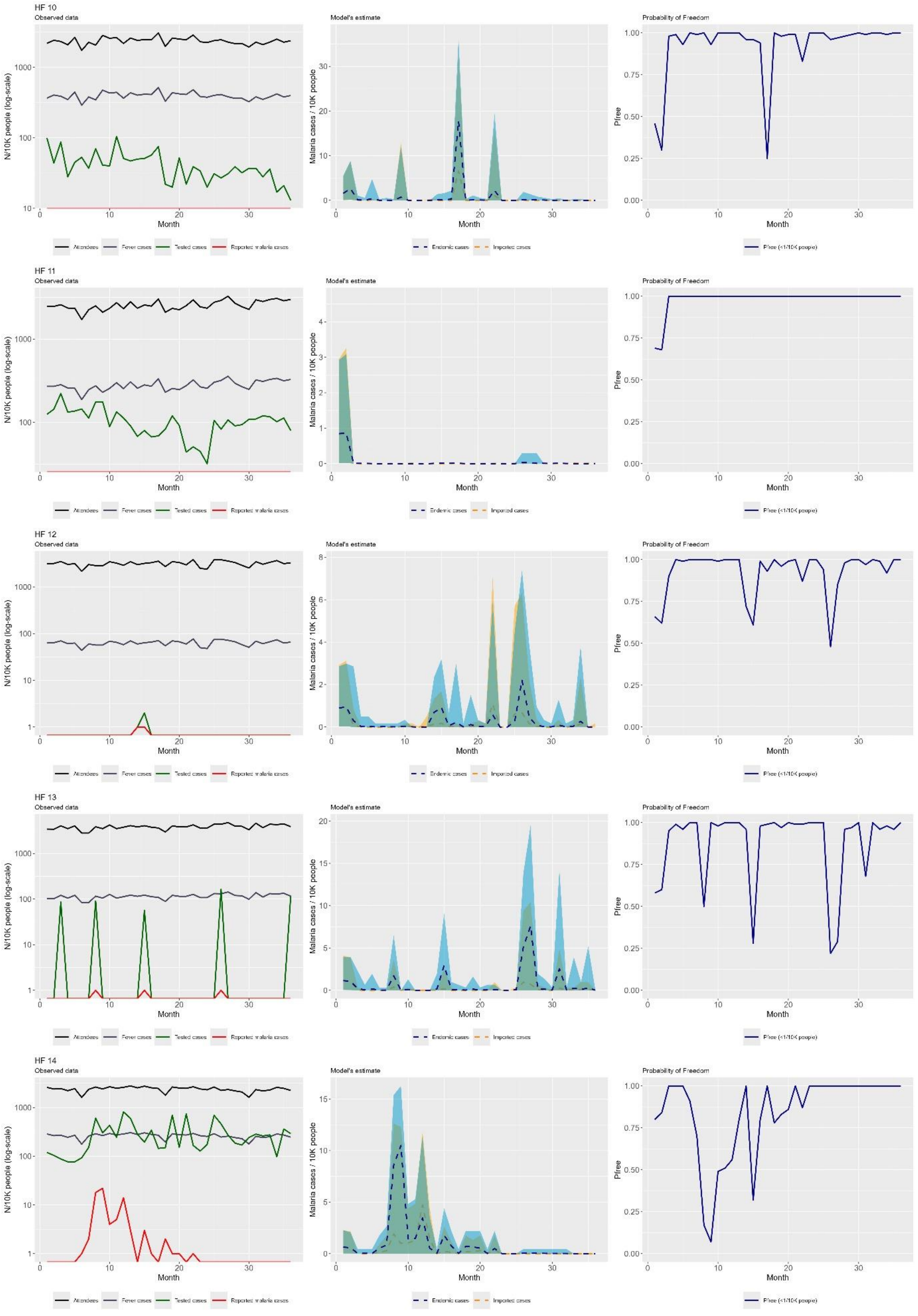

HF 10
Observed data
Model's estimate
Probability of Freedom
HF 11
HF 12
HF 13
HF 14
N/10K people (log-scale)
Malaria cases / 10K people
Pfree
Month
Attendees
Fever cases
Tested cases
Reported malaria cases
Endemic cases
Imported cases
Pfree (<1/10K people)

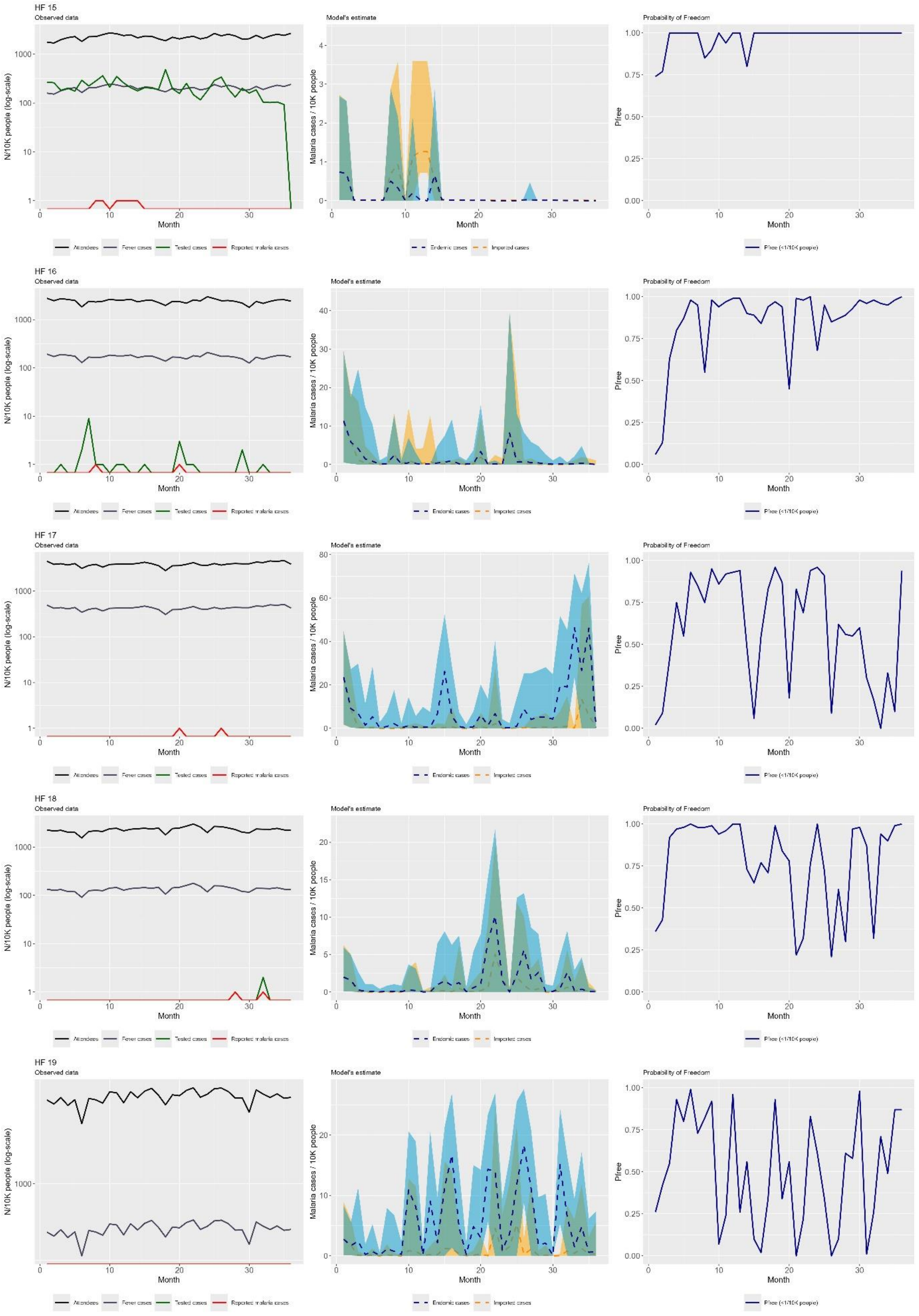
HF 15
Observed data
Model's estimate
Probability of Freedom
N/10K people (log-scale)
Malaria cases / 10K people
Pfree
Month
Attendees
Fever cases
Tested cases
Reported malaria cases
Endemic cases
Imported cases
Pfree (<1/10K people)
HF 16
HF 17
HF 18
HF 19

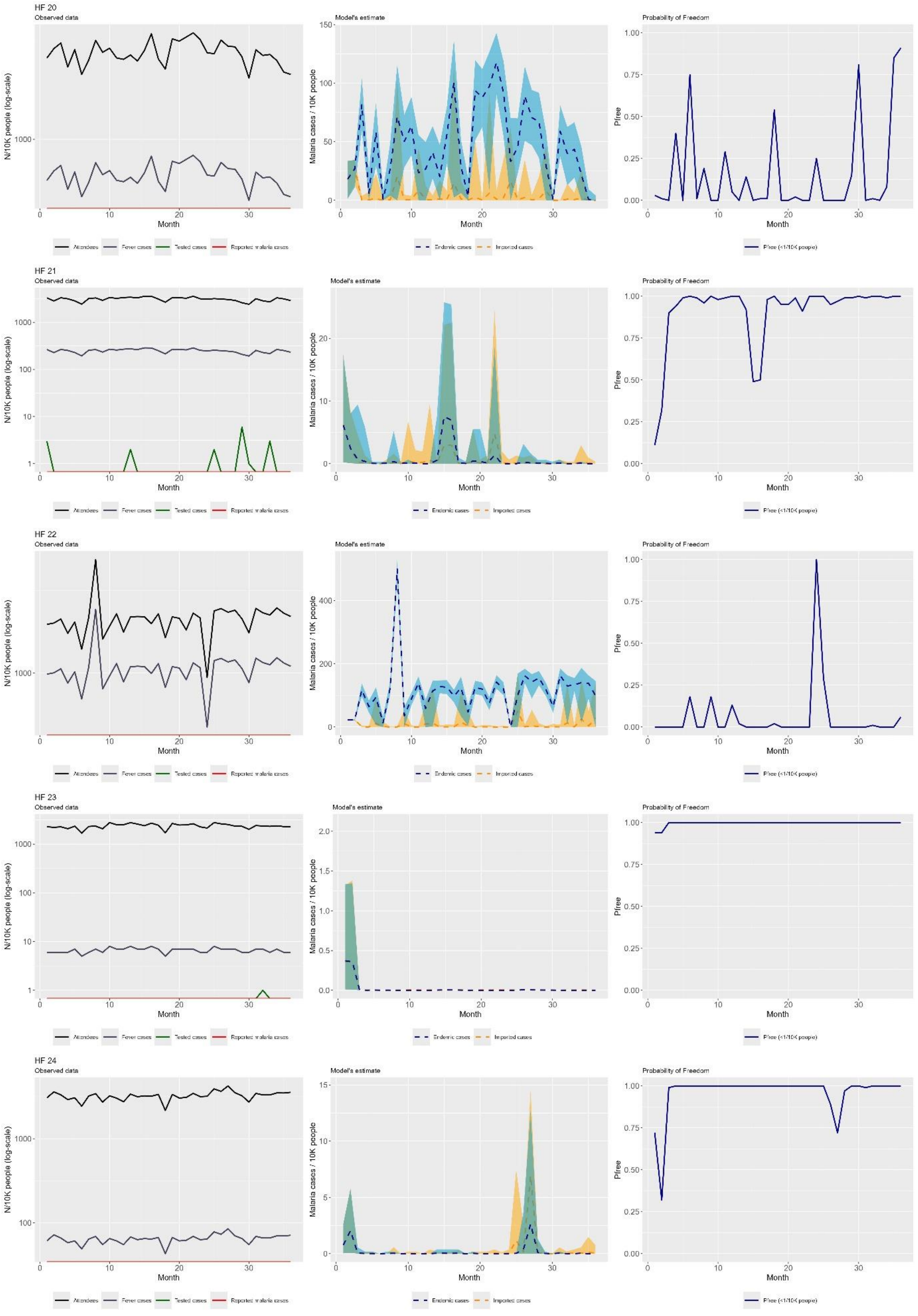
HF 20
Observed data
Model's estimate
Probability of Freedom
HF 21
HF 22
HF 23
HF 24
N/10K people (log-scale)
Malaria cases / 10K people
Pfree
Month
Attendees
Fever cases
Tested cases
Reported malaria cases
Endemic cases
Imported cases
Pfree (<1/10K people)

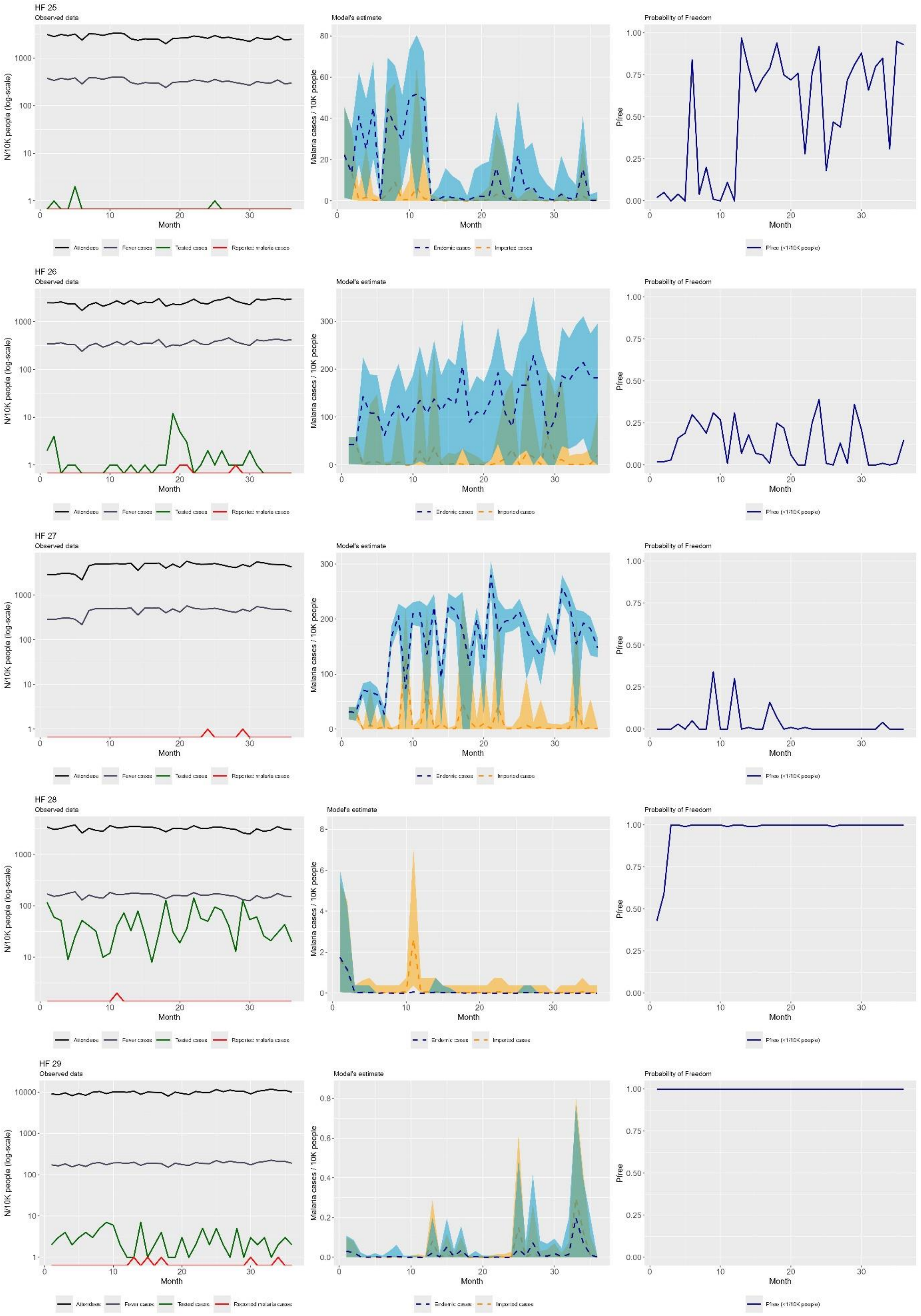
HF 25
Observed data
Model's estimate
Probability of Freedom
N/10K people (log-scale)
Malaria cases / 10K people
Pfree
Month
Attendees
Fever cases
Tested cases
Reported malaria cases
Endemic cases
Imported cases
Pfree (<1/10K people)
HF 26
Observed data
Model's estimate
Probability of Freedom
HF 27
Observed data
Model's estimate
Probability of Freedom
HF 28
Observed data
Model's estimate
Probability of Freedom
HF 29
Observed data
Model's estimate
Probability of Freedom

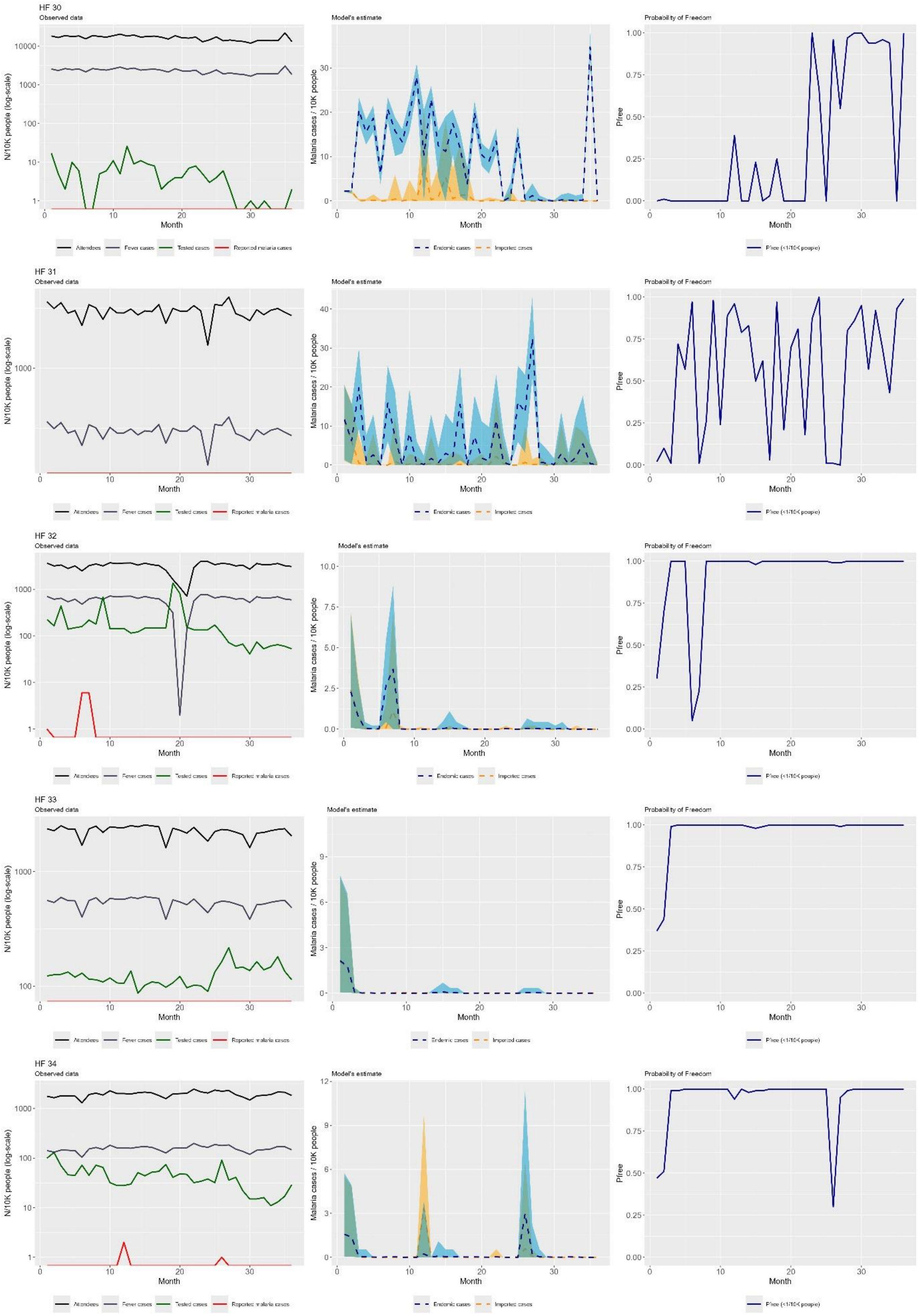
HF 30
Observed data
Model's estimate
Probability of Freedom
N/10K people (log-scale)
Malaria cases / 10K people
Pfree
Month
Attendees
Fever cases
Tested cases
Reported malaria cases
Endemic cases
Imported cases
Pfree (<1/10K people)
HF 31
HF 32
HF 33
HF 34

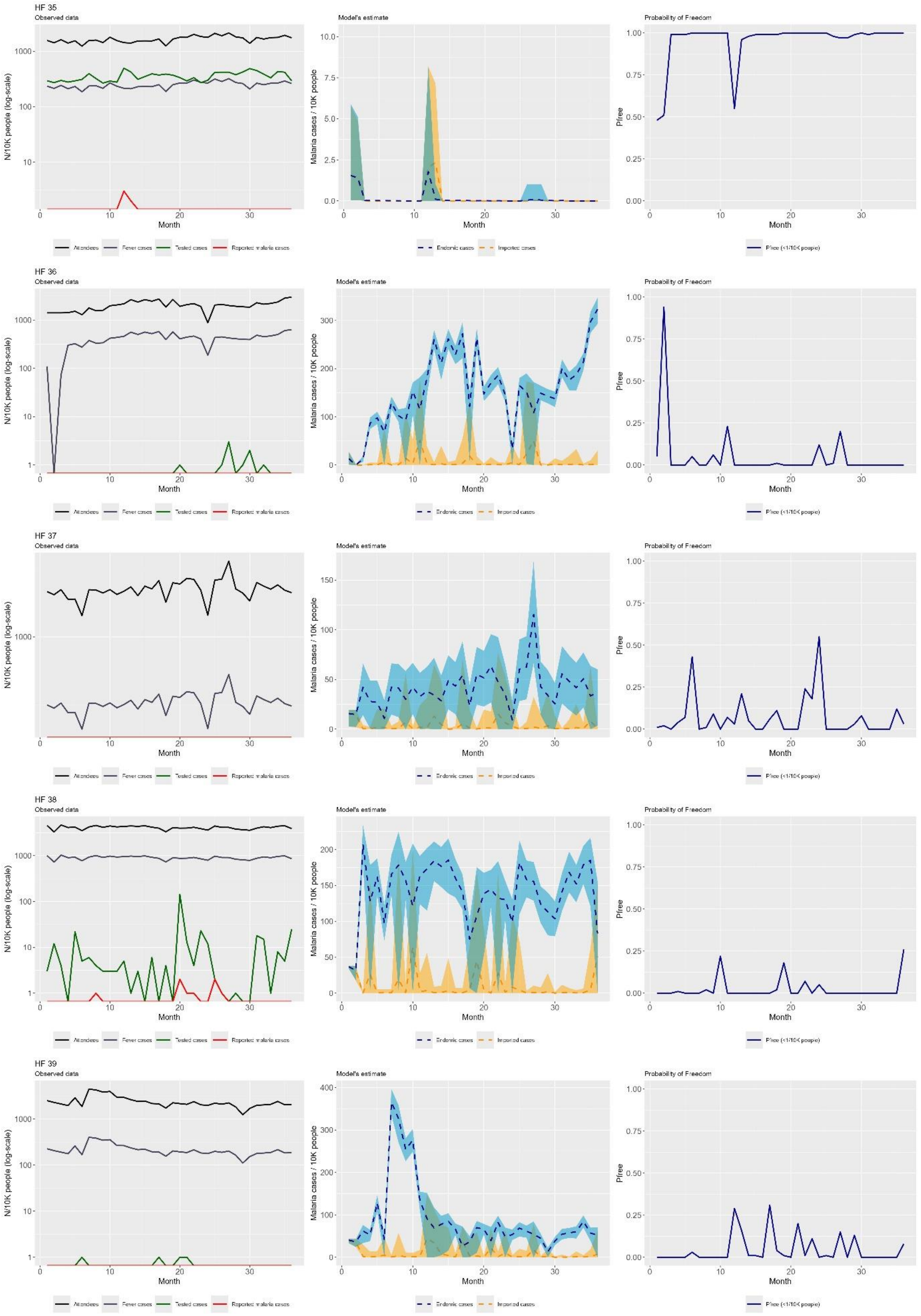
HF 35
Observed data
Model's estimate
Probability of Freedom
N/10K people (log-scale)
Malaria cases / 10K people
Pfree
Month
Attendees
Fever cases
Tested cases
Reported malaria cases
Endemic cases
Imported cases
Pfree (<1/10K people)
HF 36
HF 37
HF 38
HF 39

HF 40

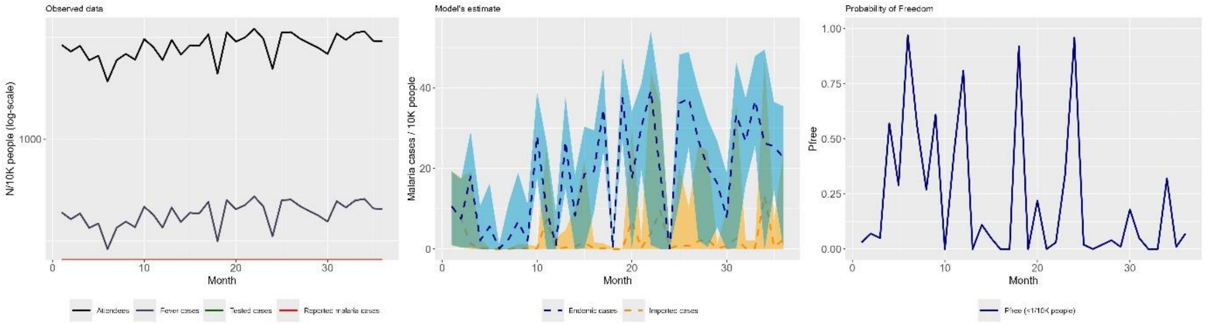

HF 41

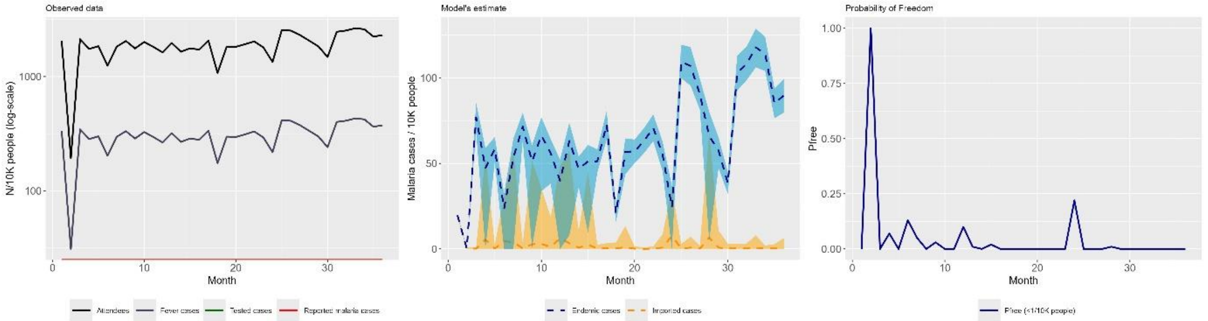

HF 42

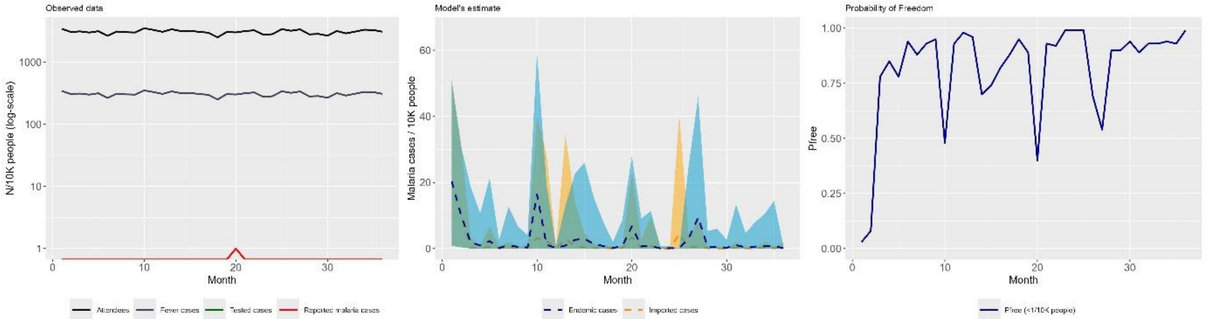

HF 43

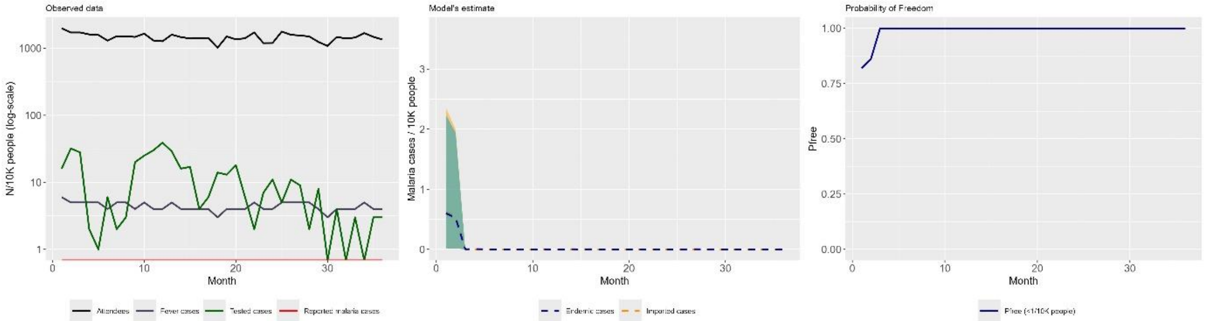

HF 44

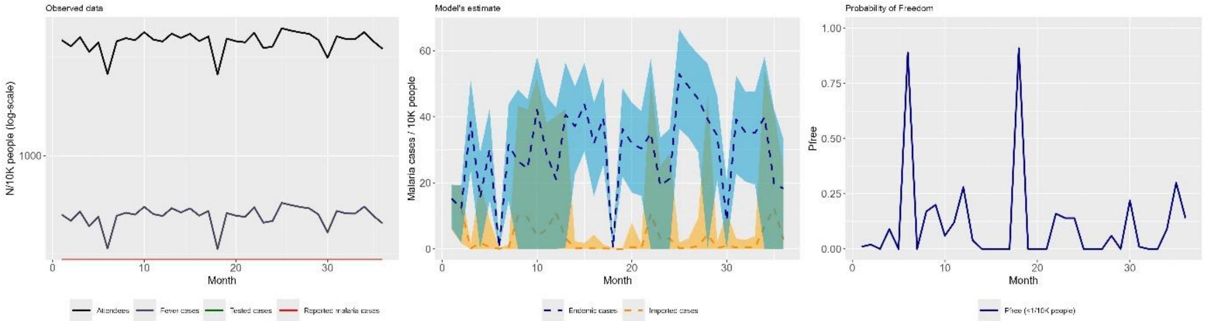

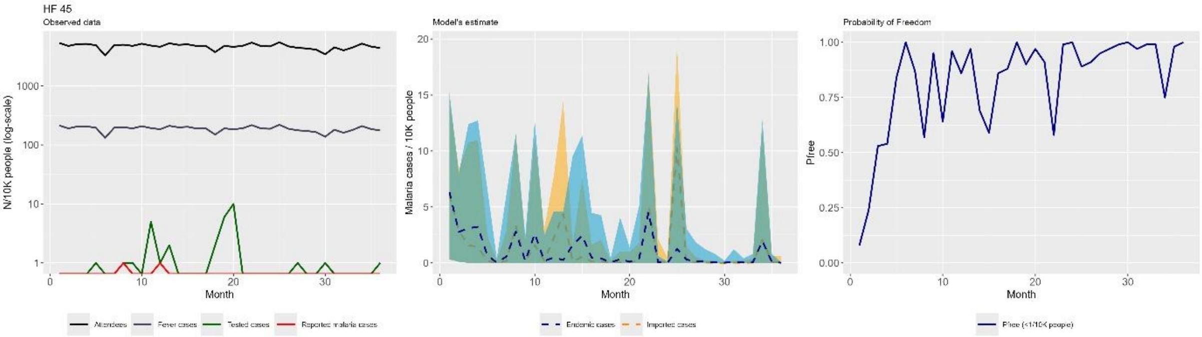
HF 45
Observed data
N/10K people (log-scale)
Month
Attendees
Fever cases
Tested cases
Reported malaria cases
Model's estimate
Malaria cases / 10K people
Endemic cases
Imported cases
Probability of Freedom
Pfree
Pfree (<1/10K people)

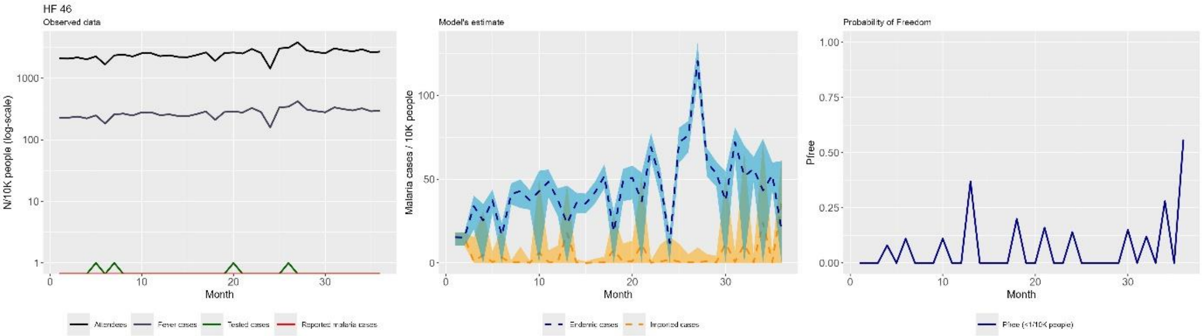
HF 46
Observed data
N/10K people (log-scale)
Month
Attendees
Fever cases
Tested cases
Reported malaria cases
Model's estimate
Malaria cases / 10K people
Endemic cases
Imported cases
Probability of Freedom
Pfree
Pfree (<1/10K people)
